# Cellular fate of a plant virus immunotherapy candidate

**DOI:** 10.1038/s42003-024-06982-0

**Published:** 2024-10-24

**Authors:** Anthony O. Omole, Jessica Fernanda Affonso de Oliveira, Lucas Sutorus, Sweta Karan, Zhongchao Zhao, Barry W. Neun, Edward Cedrone, Jeffrey D. Clogston, Jie Xu, Michael Sierk, Qingrong Chen, Daoud Meerzaman, Marina A. Dobrovolskaia, Nicole F. Steinmetz

**Affiliations:** 1 Aiiso Yufeng Li Family Department of Chemical and Nano Engineering, University of California, San Diego, La Jolla, CA USA; 2 Shu and K.C. Chien and Peter Farrell Collaboratory, University of California, San Diego, La Jolla, CA USA; 3 Center for Nano-ImmunoEngineering, University of California, San Diego, La Jolla, CA USA; 4 Moores Cancer Center, University of California, San Diego, La Jolla, CA USA; 5 Nanotechnology Characterization Lab, Cancer Research Technology Program, Frederick National Laboratory for Cancer Research sponsored by the National Cancer Institute, Frederick, MD USA; 6 Center for Biomedical Informatics and Information Technology, National Cancer Institute, National Institutes of Health, Bethesda, MD USA; 7 Department of Bioengineering, University of California, San Diego, La Jolla, CA USA; 8 Department of Radiology, University of California, San Diego, La Jolla, CA USA; 9 Institute for Materials Discovery and Design, University of California, San Diego, La Jolla, CA USA; 10 Center for Engineering in Cancer, Institute of Engineering Medicine, University of California, San Diego, La Jolla, CA USA

**Keywords:** Virology, Nanoparticles

## Abstract

Cowpea mosaic virus (CPMV) is a plant virus that is currently being developed for intratumoral immunotherapy. CPMV relieves the immune system from tumor-induced immunosuppression; reprograms the tumor microenvironment to an activated state whereby the treated and distant tumors are recognized and eradicated. Toward translational studies, we investigated the safety of CPMV, specifically addressing whether pathogenicity would be induced in mammalian cells. We show that murine macrophage immune cells recognize CPMV; however, there is no indication of de novo viral protein synthesis or RNA replication. Furthermore, we show that CPMV does not induce hemolysis, platelet aggregation and plasma coagulation amongst other assays in human blood and immune cells. Taken together, we anticipate that these results will reinforce the development of CPMV as an immunotherapeutic platform.

## Abstract (web summary)

Studies of cowpea mosaic virus (CPMV) reveals safety and non-pathogenicity of drug candidate in mammalian cells, bolstering support for intratumoral immunotherapy clinical translation.

## Introduction

As cancer continues to be a leading cause of death globally, there is a need to develop therapeutic interventions that are tumor-targeted and provide durable protection to prevent recurrence. Cancer immunotherapy takes advantage of the immune system to reverse immunosuppression and target cancer cells; if successful, long-lasting anti-tumor immunity can be achieved^1^. Of this class, the most commonly known are immune checkpoint therapies^2^, toll-like receptor agonists^3^, as well as engineered oncolytic viruses^4^. For example, Talimogene laherparepvec by Amgen (TVEC) is an FDA-approved oncolytic intratumoral immunotherapy designed to selectively lyse cancer cells and express cytokines to prime immune cell recruitment and activation, leading to tumor cell death and processing of tumor-associated antigens and neoantigens^5^. In addition to these, there are newer technologies in the development pipeline.

Cowpea mosaic virus (CPMV), a plant virus, is being developed as an intratumoral immunotherapy drug candidate. CPMV intratumoral immunotherapy reprograms the tumor microenvironment by activating and recruiting innate immune cells (particularly macrophages and neutrophils as the key contributors) that kill tumor cells and also process tumor-associated antigens and neoantigens^6,7^. This initial activation of the innate immune system then primes the adaptive immune system and leads to systemic and durable anti-tumor immunity in tumor mouse models and canine pets with cancer with observed abscopal effect^8,9^. Mechanistically, CPMV is not an oncolytic virus but rather serves as an adjuvant that possesses pathogen-associated molecular patterns (PAMPs) that are recognized by pattern recognition receptors (PRRs) such as toll-like receptors^10^. As we pave the way toward human clinical studies, a detailed understanding of the biological fate of CPMV is needed.

**Fig. 1:**
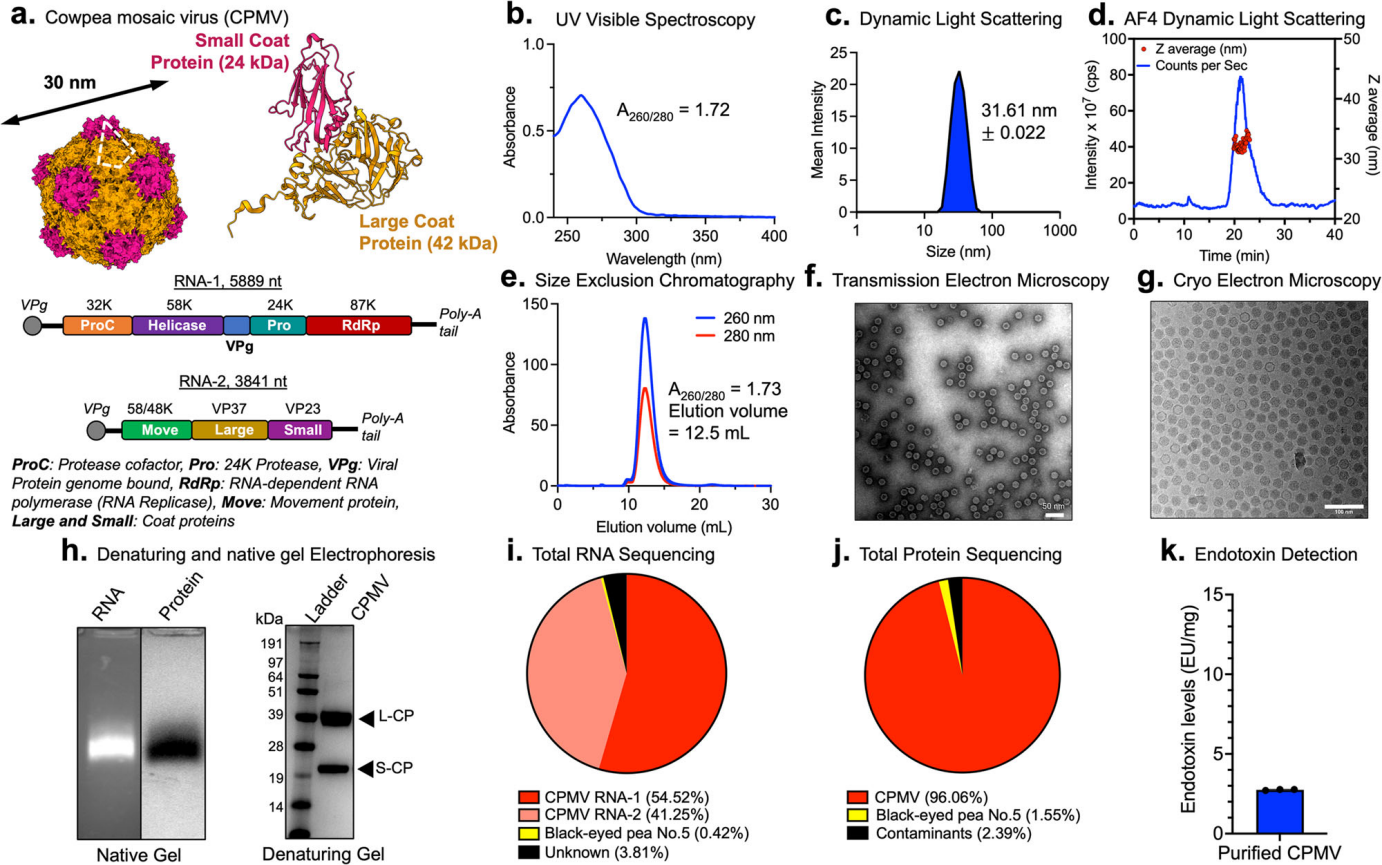
CPMV structure and nanoscale characterization. **a** CPMV structure: Cowpea mosaic virus (CPMV) has a bipartite ssRNA genome; the RNA-1 and RNA-2 are separately encapsulated into isometric 30 nm-sized capsids comprised of a small (S) and large (L) coat protein. CPMV RNA-1 encodes the 32 K protease cofactor, 58 K helicase, VPg, 24 K protease, and 87 K RNA-dependent RNA polymerase. RNA-2 encodes for the movement protein (MP), and the L and S coat proteins. PDB: 1NY7, CPMV structure generated with UCSF’s ChimeraX. **b**–**j** Nanoparticle characterization: **b** UV–VIS spectrum and the A260/280 ratio; intact CPMV have an A260/280 ratio of 1.7 ± 0.1. **c**, **d** DLS and AF4-DLS show a monodisperse ~30 nm-sized nanoparticle. **e** SEC shows the typical elution profile from a Superose 6 Increase column with a ~1.7 A260/280 ratio. **f**, **g** TEM of negatively stained CPMV and CryoEM of CPMV confirms the presence of monodisperse ~30 nm-sized CPMV. Scale bars at 50 nm and 100 nm, respectively. **h** Native gel visualized under UV light with RNA staining (GelRed) and white light after protein staining (Coomassie Blue); RNA and protein co-migrate, which indicates stable encapsulation of the RNA into the capsid. Denaturing gel: pure separations of CPMV L and S coat protein (L-CP and S-CP) at ~42 kDa and ~24 kDa, respectively. **i** Total RNA sequencing of RNA extracted from CPMV indicates 95% purity with sequences aligning with CPMV RNA-1 and RNA-2 [NC_003549.1 and NC_003550.1], 3% is unknown, and 0.4% aligns with black-eyed pea host. **j** Total protein sequencing of purified CPMV indicates 96.1% purity with sequences aligning with the CPMV coat proteins, 1.5% aligned with black-eyed pea host, and 2.4% aligned with a mixture of homo sapiens and bacterial contaminants. **k** Endotoxin detection from purified CPMV. *N* = 3.

At the cellular level, reports that dissect the fate of CPMV upon innate immune cell uptake are lacking. It is known that CPMV interacts with mammalian cells and, in fact, displays tropism towards antigen-presenting cells, similar to pathogenic animal viruses^11,12^. CPMV is endocytosed into mammalian cells in a dose and time-dependent manner in part by binding to vimentin^11,13,14^. While reports state that CPMV is non-infectious toward mammals, we could not find data supporting these claims. Thus, whether CPMV can cross kingdom borders, replicate, and translate in mammalian cells remains unknown. CPMV is a plant picornavirus with a bipartite genome (RNA-1 and RNA-2 separately encapsidated)^15^. CPMV naturally infects black-eyed peas (*Vigna uniguiculata*) and other legumes and has a narrow distribution across the country and globally^16^. Upon entry into plant host cells, CPMV RNA-1 is translated by host translation factors into a 200 kDa polyprotein that is proteolytically processed^17^. As shown in Fig. 1a, CPMV RNA-1 encodes for a 32 kDa co-factor protein involved in regulating proteolytic cleavage of RNA-1 and RNA-2 polyprotein^18^, a 58 kDa NTP/membrane binding protein with helicase activity^19^, a genome linked protein that serves as a primer for RNA replication^20^, a 24 kDa protease that processes viral polyproteins^21^, and an RNA dependent RNA polymerase (RdRp) for replicase activity^22^. RNA-1 is known to independently replicate because of the RdRp^23^; however, it relies on proteins encoded by RNA-2 for systemic transportation and systemic infection in the plant host^24^. CPMV RNA-2 has two AUG start codons and thus yields either a 105 kDa or 95 kDa polyprotein^25^. Proteolytic cleavage results in a 58 kDa/48 kDa movement protein for cell-to-cell movement^26^, and the two coat proteins, large and small, form a 30 nm capsid to encapsidate both RNAs separately (Fig. 1)^17^.

A growing body of data highlights plant virus exposure in humans; for example, pepper mild mottle virus has been identified in human stool samples, and antibodies against some plant viruses are prevalent in the human population^16,27^. We have also shown that in a human patient cohort study, plant virus antibodies can be detected in plasma (25% were positive for CPMV antibodies)^16^. While repurposing plant viruses for preclinical drug development is not a new concept, only a few have transitioned into clinical trials. For example, Folia Biotech developed a vaccine adjuvant against seasonal flu using recombinant papaya mosaic virus (NCT02188810)^28^. Kentucky BioProcessing Incorporated developed and tested a COVID19 vaccine candidate using UV-inactivated tobacco mosaic virus (NCT04473690)^29^. With the demonstrated efficacy of CPMV intratumoral immunotherapy in tumor models and canine pets with cancer, our goal is to generate a detailed understanding of CPMV in mammalian cells to pave the way into human clinical trials.

To dissect the fate of CPMV in mammalian cells, we considered a murine macrophage cell line RAW 264.7 as a model since CPMV interacts with myeloid cells, including macrophages^11,30^. First, we monitored viral entry and introduction of viral RNA upon CPMV particle exposure to macrophages. Then, we monitored the presence of the viral genome and whether there was evidence of replication or translation of viral coat proteins, viral protease, or the precursor polyprotein over 96 h post a 24 h-incubation period; this was paralleled with monitoring changes in the mammalian proteome due to plant viral introduction. In addition, we assessed the potential toxicity of CPMV to human blood and immune cells through a series of hemocompatibility and immune function assays. Findings from this study garner support for the translation of CPMV intratumoral immunotherapy toward clinical applications.

## Results and discussion

### Preparations of CPMV yield monodisperse, uniform, and pure CPMV

Being a biological nanomaterial, CPMV preparations show a high degree of quality control with monodispersed and reproducible preparations. UV-visible spectroscopy and the CPMV-specific extension coefficient are used to determine its concentration. UV–visible spectra indicate pure CPMV preparations with the characteristic A260:280 ratio average of 1.7 ± 0.1 (Fig. 1b). This confirms the presence of CPMV with RNA-1 and RNA-2^31^. Particle sizing by dynamic light scattering (DLS) and asymmetric-flow field-flow fractionation (AF4-DLS), indicate monodispersed 30 nm-sized nanoparticles with a polydispersity index of ±0.022 (Fig. 1c, d). Size exclusion chromatography shows a typical elution profile with the nucleoprotein complex eluting at 12.5 mL from the Superose6 Increase column. The characteristic A260:280 ratio is also observed, and aggregation or broken particles are not evident from the chromatogram (Fig. 1e). These data are consistent with transmission electron microscopy (TEM) and cryo-electron microscopy (CryoEM) also show a uniform distribution of nanoparticles (Fig. 1f, g). Broken particles were not observed, thereby attesting to the structural integrity of the CPMV preparations. Native gel electrophoresis indicates the comigration of RNA (stained with GelRed) and CPMV coat protein (stained with Coomassie blue). This confirms that the stained RNA is encapsulated (Fig. 1h). Denaturing gel electrophoresis of the coat proteins shows CPMV L and S coat protein (L-CP and S-CP) detected at 42 kDa and 24 kDa, respectively. Native and denaturing gels do not indicate the presence of protein or nucleic acid contaminants. To further validate the purity of the CPMV preparations and gain insights into the composition of host nucleic acid, we extracted CPMV RNA from purified particles and performed total RNA sequencing (Fig. 1i). Matching the sequences to the National Center for Biotechnology Information (NCBI) database indicates that 95.78% of the RNA sequences were CPMV RNA-1 and RNA-2, while 0.42% matched to black-eyed pea host and 3% was unmatched. In addition, we digested purified CPMV particles into peptides using trypsin, and then a solution of these peptides was analyzed using ultra-high-pressure liquid chromatography and mass spectroscopy (LC–MS/MS). Each identified peptide sequence was matched to the NCBI database (Fig. 1j). 96% of identified proteins matched with CPMV coat proteins. 1.5% of identified protein sequences match with black-eyed pea host and 2.4% of identified proteins match with a mix of common contaminants originating from humans, *Pseudomonas fluorescens*, and *Escherichia coli*. Cowpea plants are endotoxin-free, and extraction of CPMV occurs under sterile conditions. Consistently, endotoxin detection, i.e., LPS, in purified CPMV particles is well below the FDA acceptable calculated standard of 12 EU for CPMV intratumoral doses in mice tumor models^32^ (Fig. 1k). Thus, additional extraction steps are not required^33^. Together data highlight that highly uniform and pure CPMV preparations can be obtained through plant molecular farming and extraction from infected leaf tissue.

### Confocal imaging reveals that CPMV RNAs are not introduced into the cytosol upon endocytosis

While the entry pathway of many picornaviruses is not fully understood, many have investigated the rate of genome release into mammalian cells during infection^34^. Research detailing poliovirus entry and uncoating showed that poliovirus begins genome release within 10 min after entry, with the majority of the RNA released by 60 min^35^. The picorna plant virus, CPMV, is endocytosed primarily through macropinocytosis and caveolae in mammalian cells and may utilize either or other pathways as a function of virus concentration^36^. CPMV traffics through the early endosome and localizes in the lysosome^37^. However, these studies only monitored the presence of its coat proteins and not its RNAs. Because virus capsid can remain in endosomal compartments while introducing RNA into the cytosol for translation^38^, we investigated the localization of CPMV protein and RNAs.

**Fig. 2:**
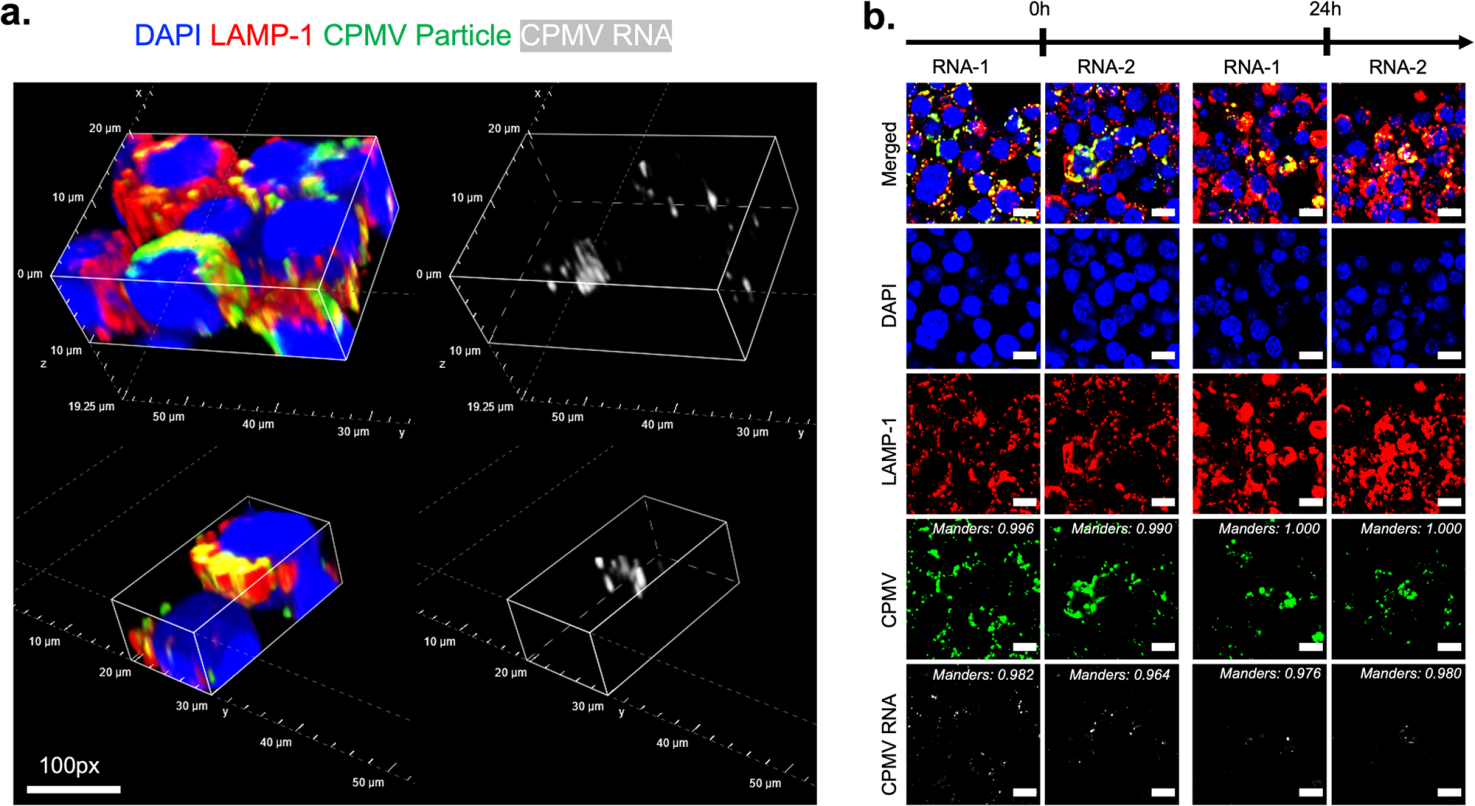
Confocal microscopy of CPMV and RNA in macrophages. **a** 3-D spatial resolution of CPMV RNAs and coat proteins colocalized with lysosomal associated membrane protein 1 (LAMP-1). Multiplexed immunofluorescence and RNA fluorescent in situ hybridization (FISH) detect CPMV coat proteins (green), RNA-1, and RNA-2 (white) colocalized with LAMP-1 (red). Scale bar to 100px. **b** Confocal images showing DAPI, LAMP-1, CPMV, and RNA channels at time points 0 and 24 h (which is 0 and 24 h additional incubation after CPMV was incubated with RAW 264.7 macrophage cells for 24 h). Fluorescent intensity of coat protein and RNAs decreases within 24 h, indicating degradation. Sustained High Mander’s Coefficient with RNA and LAMP-1 over time. At 0 h, Mander’s Coefficient with CPMV to RNA-1 and RNA-2 are 0.934 and 0.901, respectively. At 24 h, Mander’s Coefficient with CPMV to RNA-1 and RNA-2 are 0.847 and 0.878, respectively. Images taken at 60× with oil objective. Scale bar to 10 μM.

We employed RNA fluorescence in situ hybridization (FISH) with optimized protocols^39,40^ to detect CPMV RNAs at timepoints 0 and 24 h after a 24-h viral incubation period. The lysosomal compartment was stained using LAMP-1 antibodies (in red), CPMV CP was stained with Alexa 555 antibodies (green), and CPMV RNAs were probed with custom fluorescently-labeled RNA probes (white). CPMV CP and RNAs colocalize with LAMP-1 after viral incubation (Fig. 2a). After the 24-h incubation period (time point 0 h), the Mander’s coefficient between CP or RNA and LAMP-1 is 0.9. At the 24-h time point, i.e., an additional 24-h incubation after CPMV uptake, fluorescent signals for CPMV CP and RNA decreased while the Mander’s coefficient was maintained (Fig. 2b). This data suggests that CP and RNA remain in and are degraded in the lysosomal compartment. There was no indication of CPMV RNA release into the cytoplasm over the 24-h timeframe. It is interesting to note that cross-presentation of CPMV is documented in the literature: i.e., CPMV immunity entails B and T cells^6^, which would indicate cytoplasmatic processing^41^. However, this work indicates that the direct release of CPMV into the cytoplasm is not apparent. Prior work highlighted that neutrophils are primary responders that take up CPMV^7^, therefore we hypothesize that cross-presentation thus may be a phenomenon where CPMV-positive neutrophils are phagocytosed and antigens processed by macrophages^42^.

### CPMV coat proteins do not increase in mammalian cells, and 24 K protease is not detected

**Fig. 3:**
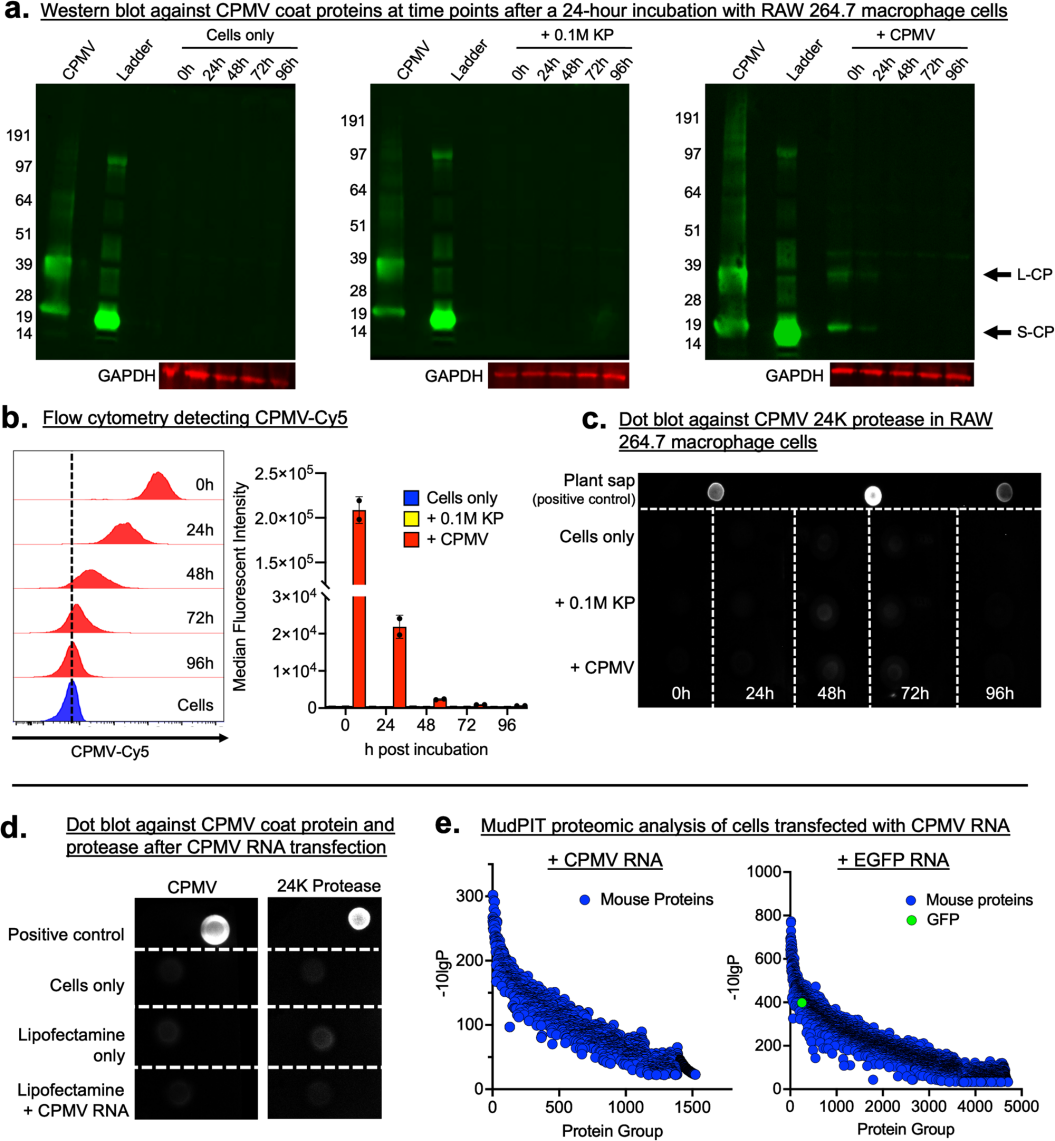
CPMV viral protein detection in mammalian cells. **a** Western blot detecting CPMV coat proteins in RAW 264.7 macrophage cells at timepoint 0 h, 24 h, 48 h, 72 h, 96 h after CPMV was incubated with RAW 264.7 macrophage cells for 24 h. L-CP and S-CP are detectable at 0 h, to a lesser degree at 24 h. GAPDH staining confirms equal concentration loading of protein lysate. Controls are negative for CPMV detection. Non-specific staining is observed at ~47 kDa. **b** Fluorescently labeled CPMV-Cy5 was incubated with RAW 264.7 murine macrophage cells and flow cytometry was used to measure CPMV levels by means of Cy5 fluorescence. Median fluorescent intensity (MFI) shows CPMV-Cy5 at 0 h time point; signals decrease over time. *N* = 2. **c** No detection of CPMV 24 K protease in RAW 264.7 macrophage cells incubated with CPMV; CPMV-infected plant sap served as positive control. This data indicates that de novo protein synthesis of the 24 K protease is not apparent in mammalian cells exposed to CPMV. **d** RAW 264.7 macrophages were transfected with CPMV RNA; both CPMV coat proteins and 24 K protease cannot be detected. Positive controls: CPMV for coat protein detection and CPMV infected plant sap for 24 K protease detection. **e** Cell lysates from RAW 264.7 macrophages transfected with CPMV RNA or a GFP expression cassette (positive control) were also analyzed by MudPIT analysis: CPMV-related proteins cannot be identified; GFP protein is detected among mouse proteins.

We investigated whether CPMV RNAs are translated by monitoring the CPMV coat protein levels as well as the 24 K protease; the latter to gain insights into de novo protein synthesis. CPMV was incubated with cells for 24 h (at 10^7^ CPMV particles per cell), and excess CPMV was removed by washing after this time point. Then longitudinal studies were performed where cells were harvested every 24 h up to 96 h, analyzed by western blot, and further quantified by flow cytometry (Fig. 3a–c). We reasoned that a steady CP signal or increase in CP signal and/or detection of the 24 K protease would be indicative of translation. Both, the L-CP and S-CP were detected at 0 h (i.e., 0 h after a 24-h exposure) (Fig. 3a, Supplementary Data 1). The CPs remained detectable at 24 h, albeit at a lower intensity when compared to the 0 h timepoint. Beyond this time point, the CPs were no longer detectable, indicating no viral protein translation. In addition, using gel-based proteomic sequencing, we confirmed the identity of CPMV coat proteins within these cells at 0 and 24 h after the 24-h incubation period. The significance of CPMV coat proteins decreases between 0 and 24 h, indicating less detection and longitudinal degradation of the CPMV protein (Supplementary Data 2 and 3). We quantified these results by flow cytometry assaying for fluorescently labeled CPMV (Cyanine 5 or Cy5) (Fig. 3b, Supplementary Data 4). Flow cytometry indicated a stepwise (10×) decrease in CPMV signal from 0 to 24 h and 24 to 48 h; at later time points, the signal is lost. Under these conditions, the results indicate no increase in CPMV CPs and, hence, viral degradation.

To assay for de novo protein synthesis, we tested for the presence of viral nonstructural proteins. Specifically, we wanted to answer whether 24 K protease was present and active—this protein is essential for viral processing and the formation of viral progeny. VP60, the precursor protein of the CPMV CPs encoded by RNA-2, is processed by the 24 K protease which is translated by RNA-1 (Fig. 1a). While 24 K protease translation is evident in infected plant tissue, 24 K protease could not be detected when CPMV was incubated with RAW 246.7 macrophage cells (Fig. 3c). Taken together, this data does not provide any evidence of viral protein translation.

The translation of CPMV and other plant viral RNA in animal cells under experimental conditions has been explored previously by others. CPMV RNA-2 was translated into its 105 and 95 kDa polyprotein when microinjected in *Xenopus Laevis* oocytes^43^. Animal cell-transient expression systems (co-transfection of a construct that encodes a T7 RNA polymerase and a construct that encodes the  gene of interest into mammalian cells) have also been used to translate plant viral RNA in mammalian cells. This system has been used by Lomonossoff and others to show the translation of 105 and 95 kDa polyprotein after transfection of a designed CPMV RNA-2 DNA plasmid into mammalian BSC-40 cells^25^. This technique has also been used to study the translation of crucifer-infecting tobamovirus^44^ and hibiscus chlorotic ringspot virus^45^ in mammalian HeLa and COS-7 cells, respectively.

While these experimental conditions do not reflect the natural process of uptake and cellular processing, we assayed whether CPMV CPs and 24 K protease could be detected upon lipofectamine-assisted CPMV RNA transfection. Confocal imaging multiplexed with RNA FISH indicated that upon transfection the CPMV RNA was not co-localized with LAMP-1 (Supplementary Movie 1). GFP RNA transfected using the same method was effectively translated but CPMV RNA was not. In protein lysates, however, we did not detect either CPMV coat protein or 24 K protease through a dot blot assay (Fig. 3d). We also performed proteomic analysis with Multidimensional Protein Identification Technology (MudPIT) to detect any CPMV proteins, including the polyproteins—this overcomes the technical hurdle that the antibodies only detected processed proteins. There was no indication of CPMV viral protein in MudPIT analysis, and there was also no indication of a new protein band in a high molecular weight denaturing gel. As a positive control, an enhanced GFP mRNA (EGFP) was also transfected, and indeed GFP was detected in gel and through mass spectrometry analysis (Fig. 3e, Supplementary Data 5a, b). In addition, we designed an in vitro transcribed (IVT) CPMV RNA for transfection by replacing a GFP ORF cassette with CPMV ORFs (Supplementary Data 5c, d). Under these conditions, the translation of IVT CPMV RNAs was also undetectable upon transfection (Supplementary Data 5e). We speculate that the CPMV IRES may not be able to recruit mammalian ribosomal units and initiation factors for RNA translation.

### Viral recognition: the presence of CPMV in RAW 264.7 macrophage cells launch anti-viral immune programming

**Fig. 4:**
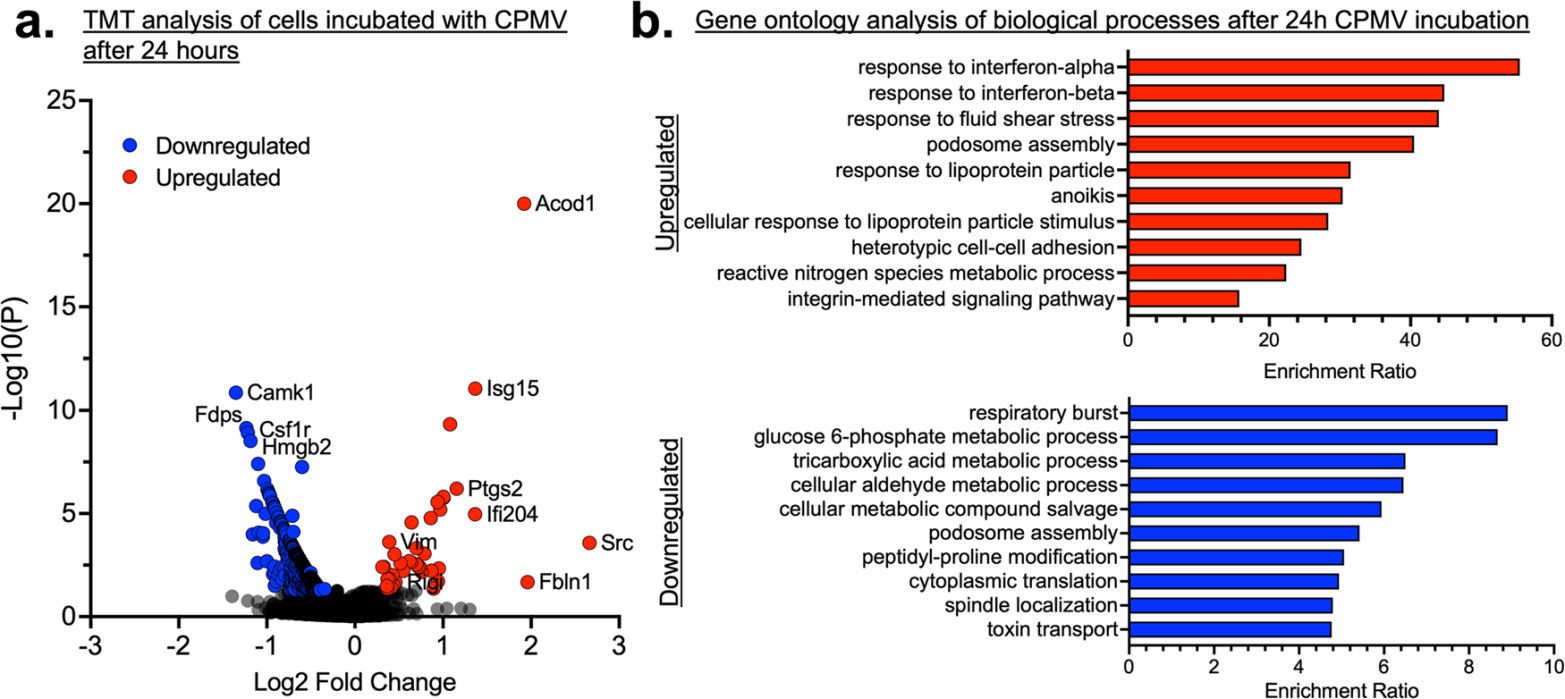
Viral recognition in mammalian cells. **a** TMT proteomics shows a volcano plot of RAW 264.7 macrophage proteome after incubation with CPMV for 24 h vs control. Log2 fold change of upregulated (red) and downregulated (blue) proteins. **b** Top 10 upregulated and downregulated biological processes after CPMV incubation.

CPMV is an immunomodulatory agent, and intratumoral therapy primes innate immune cell activation with type I IFN signaling^10^. Here, we used a TMT spectrometry approach to delineate the proteomic response to CPMV, i.e., upregulation and downregulation of proteins at 0 h after 24-h incubation (Fig. 4a). There are more downregulated proteins than there were upregulated (a full list of upregulated and downregulated proteins are described in Supplementary Table 1 attached). Out of the proteins identified, we found that CPMV induced the upregulation of antiviral-related proteins, including interferon-stimulated gene-15 (*ISG15*), interferon-induced protein 44, immunity-related GTPase M1, interferon-activable protein 204, and retinoic acid-inducible gene (*RIG-1*). Vimentin, a known binder to CPMV^11,13,14^, is also upregulated 24 h after CPMV incubation. Vimentin can act as a sensor and co-receptor for viruses and facilitate endocytosis and assembly in the cytosol^46^. In addition, studies have also shown that vimentin plays a role in immune regulation^47,48^. We are currently studying how this is related to CPMV’s mechanism of action. Once viruses are recognized in mammalian cells, antiviral signaling is launched, causing the upregulation of interferon-stimulated genes like *ISG15* and interferon-induced protein with tetratricopeptide repeats 1 (*IFIT1*) to combat infection^49,50^. *ISG15*, in particular, is rapidly upregulated during viral infection and has been implicated with antiviral activity towards animal picornaviruses such as foot and mouth disease virus (FMDV), coxsackievirus B3 (CV-B3) and Seneca virus A (SVA)^51^. On the other hand, we found that colony-stimulating factor 1 receptor (*CSF1R*), a receptor associated with poor tumor prognosis and immunosuppression^52^, was downregulated. Indeed, inhibitors against *CSF1R* are under clinical development as anti-cancer therapeutic agents^52^. We also performed gene ontology analysis of the proteins being upregulated and downregulated (Fig. 4b, Supplementary Table 1). The majority of upregulated proteins correspond with interferon-related processes after CPMV exposure. While interferon signaling is primarily an antiviral response, it also possesses anti-tumor functions^53^. Indeed, interferon signaling has been shown to play a role in the unique potency of CPMV intratumoral immunotherapy^54^. Interestingly, many downregulated processes are metabolic, perhaps to mitigate viral spread. Taken together, this data supports the immunomodulatory nature of CPMV and highlights its interactions with innate immune cells—CPMV launches an anti-viral and immune-activated program, which in the context of intratumoral immunotherapy elicits potent antitumor immunity^10^.

### No evidence of RNA replication in innate immune cells

**Fig. 5:**
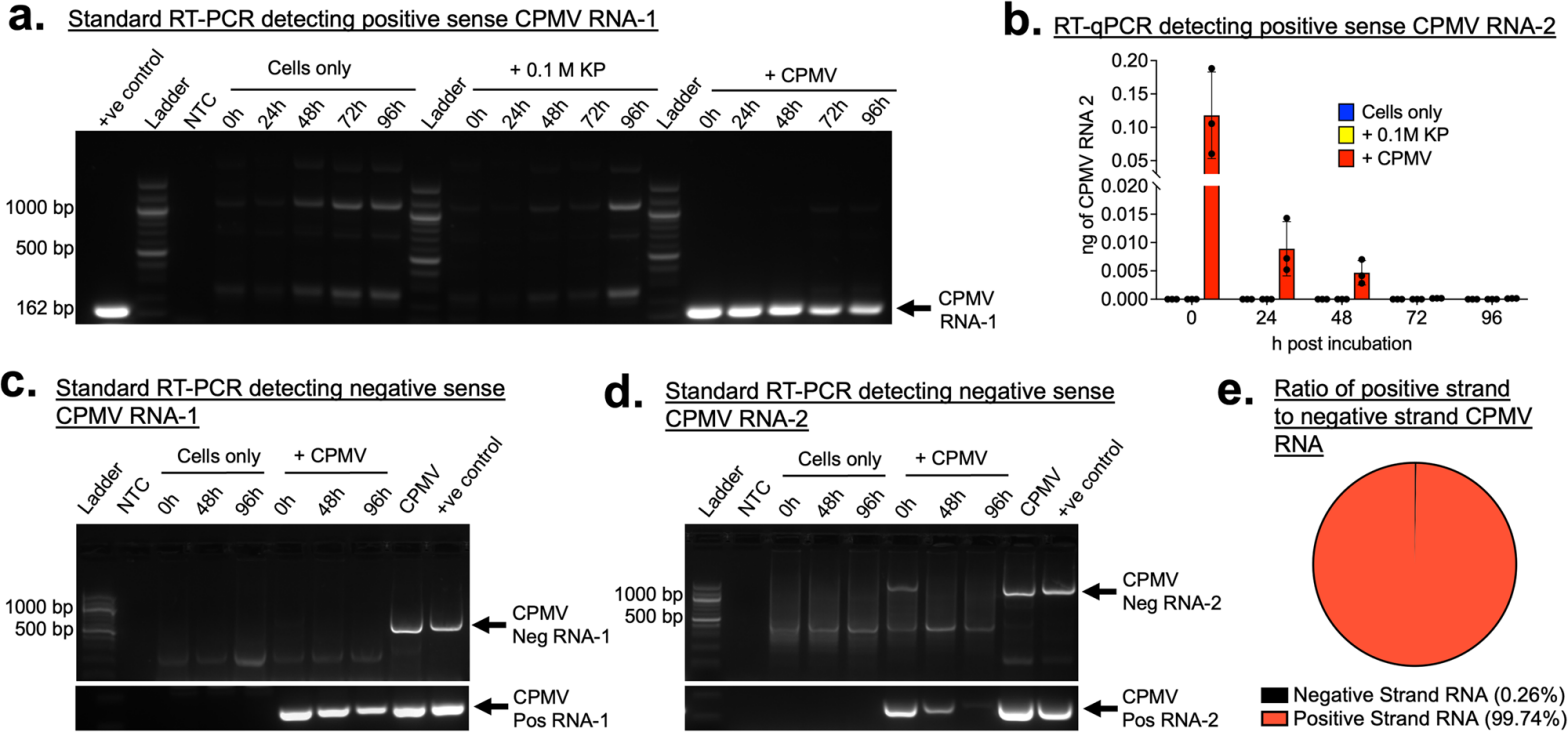
CPMV viral RNA detection in mammalian cells. **a** Standard RT-PCR identifies CPMV RNA-1 at 162 bp and decreases over 0, 24, 48, 72, and 96 h after CPMV was incubated with RAW 264.7 macrophage cells for 24 h. No detection of CPMV RNA-1 in control samples. **b** Quantitative RT-PCR shows a decrease in CPMV RNA-2 and no detection in control samples over 0 h, 24 h, 48 h, 72, 96 h after CPMV was incubated with RAW 264.7 macrophage cells for 24 h. *N* = 3. **c** Negative strand CPMV RNA-1 cannot be detected in samples with positive stand CPMV RNA-1 over 0 h, 48 h, 96 h after CPMV was incubated with RAW 264.7 macrophage cells for 24 h. A positive control is total RNA extracted from CPMV-infected leaves. **d** Negative strand CPMV RNA-2 cannot be detected in samples with positive stand CPMV RNA-2 over 0, 48, and 96 h after CPMV was incubated with RAW 264.7 macrophage cells for 24 h. A positive control is total RNA extracted from CPMV-infected leaves. **e** Total RNA sequencing of pure extractions of CPMV shows 99% align with positive-strand CPMV RNA and 0.26% align with negative-strand CPMV RNA.

In tandem with viral protein translation, we probed whether CPMV RNAs were being replicated. Using reverse transcriptase polymerase chain reaction (RT-PCR), we probed the amounts of CPMV RNA-1 and RNA-2 in RAW264.7 macrophage cells at 0 h through 96 h after a 24-h incubation with CPMV. Under these conditions, standard RT-PCR indicates a decrease in CPMV RNA-1 identified at 162 base pairs (Fig. 5a), and quantitative RT-PCR also indicates a decrease in CPMV RNA-2 (Fig. 5b, Supplementary Data 6a, b). Because RNA-1 encodes for the RdRp, once translated this RNA can independently replicate^23^. Since we did not observe the increasing amounts of CPMV RNA-1, these results suggest that it is neither being translated into viral protein nor replicated.

This finding is in agreement with the fate of TMV RNA in bone marrow-derived macrophages. Though TMV was found to be endocytosed in these cells, a decrease in RNA copies was observed over two weeks, indicating degradation^55^. However, there are reports that suggest a small number of plant viruses can cross kingdoms and be replicated and/or translated in their insect vectors^56^. An example is tomato spotted wilt virus (TSWV) which has been shown to replicate in *Frankliniella occidentalis* insect cells^57^. Others have shown that TSWV can express proteins in human cell lines under experimental conditions^58^. Thus, to probe this further, we focused on the positive and negative strands of the viral RNA. Positive strand viral RNA replication involves the synthesis of negative-sense RNAs, and so detection of negative-strand RNA would confirm viral replication^27^. Using tagged RT-PCR probes^59,60^, we investigated the presence of negative strand CPMV RNAs in RAW 264.7 macrophage cells at 0, 48, and 96 h after a 24-h viral incubation. As positive controls, total RNA was isolated from CPMV-infected plant tissue. Standard RT-PCR indicates that negative strand CPMV RNA-1 is not detected at any timepoint even though these cells all had positive strand CPMV RNA-1 (Fig. 5c, Supplementary Data 7). However, we detected negative strand CPMV RNA-2 only at 0 h and not at 48 and 96 h though positive strand CPMV RNA-2 was present (Fig. 5d, Supplementary Data 7). We also detected negative strand CPMV RNAs from total RNA isolated from the purified CPMV particles, and so we performed total RNA sequencing on isolated RNA. As expected, while the majority of identified RNA sequences matched with positive-strand CPMV RNAs, about 0.26% aligned with negative-strand, which is likely an artifact of contamination during CPMV extraction from infected black-eyed pea host. Taken together, data do not suggest RNA replication but rather indicate degradation of the RNAs.

### CPMV is hemocompatible in human blood and does not affect the functionality of human immune cells

**Fig. 6:**
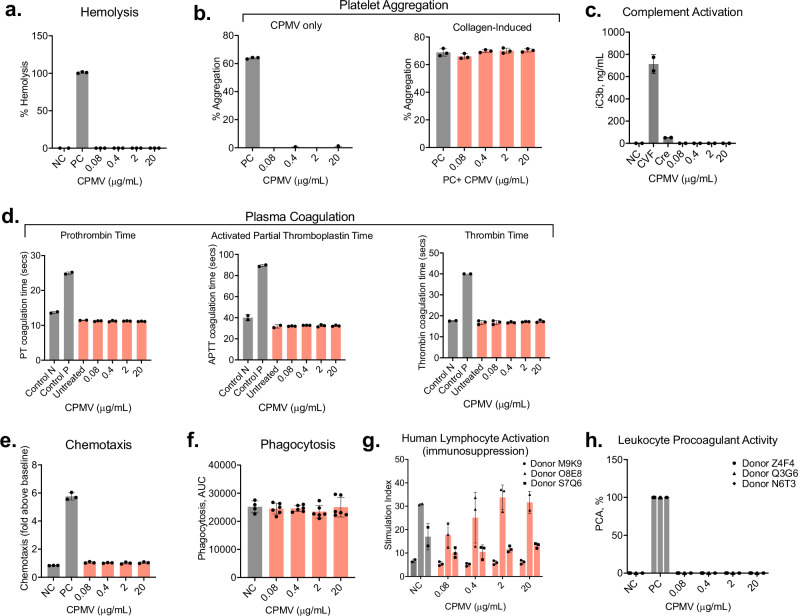
CPMV demonstrates hemocompatibility in healthy human blood and does not negatively affect the function of human immune cells in vitro. **a** Hemolysis was tested in human whole blood. PBS and Triton X-100 were used as negative control (NC) and positive Control (PC), respectively. *N* = 3. **b** Platelet aggregation was assessed in platelet-rich plasma. Collagen was used as a positive control (PC). *N* = 3. **c** Complement activation was not detected in human plasma in the presence of CPMV. PBS and cobra venom factor were used as negative (NC) and positive (PC) control, respectively. Cremophor-EL (Cre) at the same concentration as in the clinical formulation Taxol was used as an additional nanoparticle-relevant positive control. *N* = 2. **d** Plasma coagulation was assessed in the presence of CPMV using prothrombin time (left graph), activated partial thromboplastin time (middle graph), and thrombin time (right graph) tests. Control N and Control P represent WHO-standardized normal and abnormal plasma, respectively. Untreated sample refers to the same healthy human donor plasma as that used for CPMV treatment. *N* = 3. **e** Chemotaxis of human myeloid cells HL-60 was studied toward PBS (negative control (NC)), serum (positive control (PC)), or CPMV. N = 3. **f** CPMV effects on phagocytosis of Zymosan A by myeloid cells HL-60 was studied. PBS was used as negative control (NC). Data are presented as an area under the curve (AUC) obtained for each study sample. *N* = 6. **g** Flu antigen-specific proliferation of human primary T-lymphocytes isolated from three healthy donors (M9K9, O8E8, and S7Q6) was not affected by the presence of CPMV. PBS was used as negative control (NC). *N* = 3. **h** Leukocyte Procoagulant Activity (PCA) was not induced by CPMV at all tested concentrations in peripheral blood mononuclear cells isolated from three healthy donors (Z4F4, Q3G6, and N6T3). PBS and a combination of *E. coli* K12 LPS and calcium ionophore were used as negative control (NC) and positive control (PC), respectively. *N* = 3.

CPMV is administered intratumorally for the intended therapeutic application, however a small yet detectable amount of the CPMV dose will leach from the tumor resulting in systemic exposure^16^. Therefore, we assessed hemocompatibility of CPMV using human whole blood and its derivatives. Under in vitro conditions, CPMV at all tested concentrations did not induce hemolysis, platelet aggregation, and complement activation and did not affect collagen-induced platelet aggregation and normal plasma coagulation time (Fig. 6a–d). In all immune function tests, CPMV did not affect the normal phagocytic function of myeloid cells and flu-antigen-specific proliferation of T-lymphocytes and, did not induce chemotaxis and leukocyte procoagulant activity (Fig. 6e–h). CPMV was tested at four concentrations, including 20 μg/mL, which is ten-fold higher than the maximum CPMV concentration expected in the blood at the intended therapeutic dose, even when this entire dose was distributed systemically after the intratumoral administration. Collectively these data imply that CPMV does not affect the integrity of erythrocytes, is not pro-thrombogenic, has a negligible risk of complement activation-related pseudoallergy (CARPA), and does not affect normal function of human leukocytes. Hematology assays used in this study demonstrated good in vitro-in vivo correlation^61^ therefore, the risk of CPMV-mediated hemotoxicity in vivo is low.

## Conclusion

**Fig. 7:**
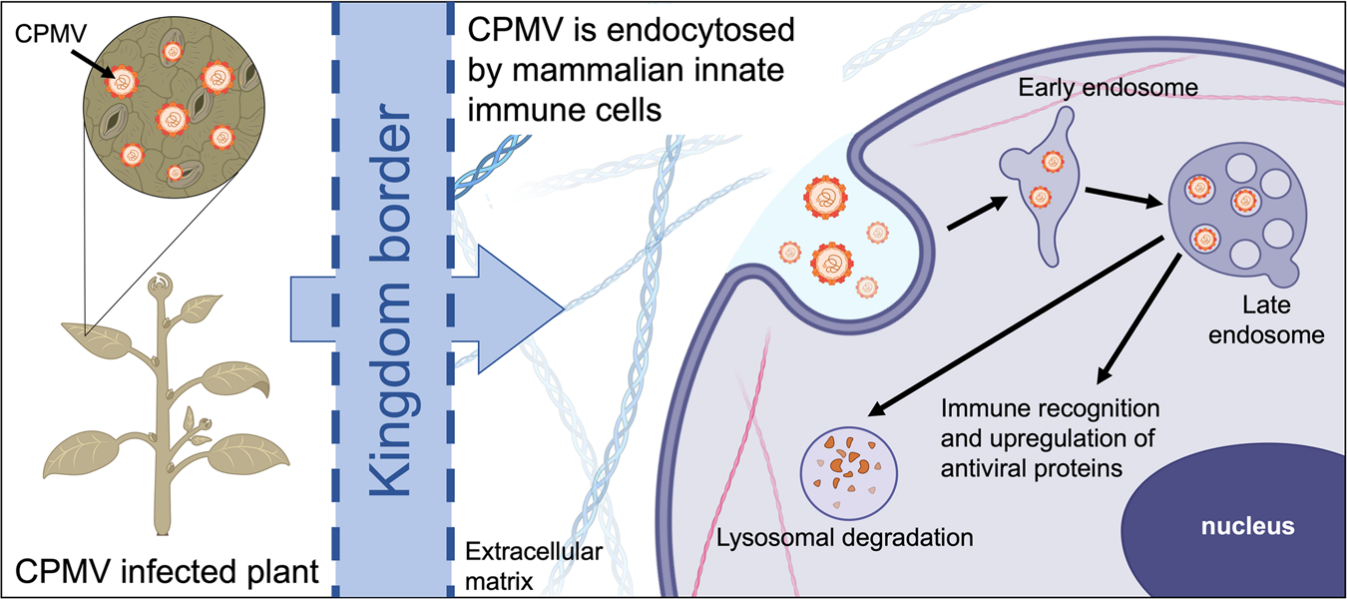
Cellular fate of CPMV schematic. CPMV, a plant virus, is endocytosed by mammalian cells and traffics to the early and late endosomes. Upon recognition, CPMV programs an antiviral immune response. CPMV capsid and RNA remain in the endolysosome, where degradation occurs. Created with BioRender.com.

Our findings in this work indicate that CPMV is endocytosed by mammalian cells into the endolysosome, where both capsid and RNA co-localize for days without the introduction of RNA or protein into the cytosol (Fig. 7). Furthermore, levels of viral capsid protein and RNA decrease over time, indicative of degradation. There are no signs of viral RNA replication or viral protein translation. However, CPMV is recognized which launches immune-related, defense, and antiviral biological processes. A limitation to the translational impact of this study is in the utility of murine RAW 264.7 macrophage cell lines. While this cell line is used in the field as a model for experiments in mammalian systems, we sought to bolster the translational impact of this study by conducting safety and toxicology studies using human blood and cells from healthy donors. These studies highlight that CPMV is non-toxic and non-cytolytic. Taken together, these results reinforce CPMV as a safe platform for intratumoral immunotherapy.

## Materials and methods

### CPMV propagation, extraction, and purification

A USDA PHQ526 permit is required to work with plant viruses. 7-day-old no. 5 Cowpea plants (*Vigna unguiculata)* were mechanically inoculated with carborundum and 2 μg of 0.1 mg/mL CPMV in 0.1 M potassium phosphate buffer pH 7. Fourteen days post-infection, mosaic infection patterns were present on the leaves. Infected leaves were harvested and stored in −80 °C until virus extraction. CPMV extraction and purification were carried out with established protocols^62^. Using N-hydroxy succinimide (NHS) chemistry, NHS-sulfo-Cy5 (Lumiprobe) was conjugated to surface lysines on CPMV’s capsid^63,64^.

### CPMV characterization

Ultraviolet–visible spectroscopy (UV–VIS), Native and denaturing gel electrophoresis, FPLC, DLS, and TEM were carried out using established protocols^16^.

#### Endotoxin detection

Endotoxin quantification on CPMV preparation was measured using Pierce™ Chromogenic Endotoxin Quant Kit (Thermo Fisher Scientific) following the manufacturer’s protocol.

#### RNA sequencing

In total, 1 μg of RNA extracted from purified CPMV particles was submitted to UCSD’s Institute of Genomic Medicine for processing. The sample was used to generate a sequencing library with Illumina® Stranded RNA kit (Illumina, San Diego, CA).

#### Protein sequencing

Purified CPMV particles were submitted to the UCSD Biomolecular and Proteomics Mass Spectrometry Facility for sequencing.

#### Cryo-transmission electron microscopy (cryo-TEM)

Stock solutions (3 µL) were applied to a glow-discharged lacey carbon film grid (Electron Microscopy Sciences) and vitrified utilizing an FEI Vitrobot plunge freezer at 95% humidity, with a blot time of 3 s and a blot force of −5. Images were taken using T20 TEM (ThermoFisher) at 200 kV acceleration voltage.

#### Asymmetric-flow field-flow fractionation (AF4)

The AF4 system consisted of an isocratic pump (Agilent G1310A, Palo Alto, CA), well-plate autosampler (Agilent G1329A), AF4 separation channel (AF4, Wyatt Technology, Santa Barbara, CA), and a dynamic light scattering (Malvern Zetasizer Nano ZS) instrument. The separation channel had a length of 275 mm and a 350 µm spacer. A regenerated cellulose membrane (10 kDa) was used for particle separation. The elution profile consisted of a cross-flow of 2 mL/min for 25 min. The detector flow was 1 mL/min, and the injection volume was 100 µL. The mobile phase was PBS (Hyclone) which was filtered through a 0.2 µm regenerated cellulose membrane prior to use. Samples were diluted 10-fold in PBS prior to injection. A Malvern Zetasizer Nano ZS instrument (Southborough, MA) with a backscattering detector (173°) was used for measuring the hydrodynamic diameter in flow mode. Measurements were made in a quartz flow cell (Malvern ZEN0023), and data was collected using Malvern Zetasizer software (v7.11). The intensity threshold was set to 400 kcps.

### Cell culture and CPMV incubation

RAW 264.7 macrophage cell line (ATCC) was cultured in 12-well plates with Dulbecco’s modified Eagle’s medium DMEM (Corning, 10-017-CV), 10% Fetal Bovine Serum (Cytiva, SH30071.02) and 1% penicillin–streptomycin (Cytiva, SV30010) at 37 °C. In total, 10^7^ CPMV particles per cell were incubated with cells for 24 h and then washed three times with 1× phosphate buffer solution (Corning, 21-040-CV). Fresh DMEM was added to the cells and maintained until harvest. At each harvest time point, cells were harvested with a cell scraper, washed with 1× PBS, and pelleted at 10,000×*g* (4 °C, 10 min). Cell pellets were stored at −80 until protein and RNA extraction.

### Protein extraction

#### From cells

Frozen cells were lysed using Pierce™ RIPA Buffer (Thermo Scientific, 89901) and 1× Halt™ Protease and Phosphatase Inhibitor Cocktail (Thermo Fisher, 78440) for 5 mins on ice, then centrifuged at 16,000×*g*, 4 °C, 15 min. Supernatant was collected and protein concentration was determined by Pierce™ BCA Protein Assay Kit (Thermo Scientific, 23227).

#### From plant sap

CPMV-infected leaves were frozen under liquid nitrogen and then ground with a mortar and pestle until they had a fine texture. Crushed leaves were resuspended in 0.1 M potassium phosphate buffer (pH 7) and filtered through a layer of Mira cloth. This solution was centrifuged to pellet leaf debris. The supernatant was isolated, and 1× Halt™ Protease and Phosphatase Inhibitor Cocktail (Thermo Fisher, 78440) was added.

#### Western Blot and dot blot

In total, 50 μg of protein lysate was loaded with 4× lithium dodecyl sulfate sample buffer (Life Technologies) and 10× NuPAGE sample reducing agent (Invitrogen) before denaturing at 95 °C for 5 min. Samples were run on 4–12% SDS-PAGE gel in 1× MOPS buffer (Thermo Scientific) at 200 V, 120 mA, 25 W. The protein lysate was then electro-transferred to a nitrocellulose membrane (Amersham Protran Premium 0.45 μm nitrocellulose, GE Healthcare). The membrane was blocked with 5% BSA in 1× PBS for 1 h and then rinsed twice with 1× PBS with 0.1% tween. For primary antibody incubation, 1 μg antibody/1 mL solution of Rabbit anti-CPMV (Pacific Immunology, 12273/12274) was incubated in 5% BSA in 1× PBS with 0.1% tween for 1 h and rinsed. For protease detection, 1:500 anti-24K protease (generously provided by Professor George Bruening) was used. For secondary incubation, 0.2 mg antibody/ 1 mL solution of Goat anti-Rabbit Alexa 555 (Invitrogen) was incubated in 5% BSA in 1× PBS with 0.1% tween for 1 h and rinsed. The membrane was imaged using ProteinSimple FluorChem R. In total, 50 μg protein was dotted on the membrane, and procedures were repeated. The membrane was developed with Pierce™ ECL Western Blotting Substrate and imaged using ProteinSimple FluorChem R. Mouse anti-GAPDH was used as loading controls, with Goat anti-Mouse Alexa 647 (Invitrogen) on the same blots.

#### Flow cytometry

In total, 10^7^ CPMV-Cy5 particles per cell were incubated with cells for 24 h and then washed three times with 1× Phosphate Buffer Solution (Corning, 21-040-CV). Fresh DMEM was added to the cells and maintained until harvest. At each timepoint, cells were harvested with a cell scraper, washed with Gibco™ Cell Dissociation Buffer, and pelleted at 500×*g* for 10 min. Cells were fixed in 4% PFA in 1× PBS for 10 min at RT washed with 1× Phosphate Buffer Solution (Corning, 21-040-CV), and pelleted at 500×*g* for 10 min. Cells were resuspended in 200 μL of Flow Cytometry Staining Buffer (2% (v/v) FBS and 0.09% (w/v) NaN_3_ in 1× PBS). Cells were run using BD Accuri C6 Plus flow cytometer (BD Biosciences). Gating and analysis were performed using FlowJo software.

#### Mass spectrometry gel band analysis

In total, 40 μg of protein lysate was added run on 4–12% SDS-PAGE gel in 1× MOPS buffer (Thermo Scientific) at 200 V, 120 mA, 25 W. Protein bands at 42 and 24 kDa were cut out and submitted to UCSD Biomolecular and Proteomics Mass Spectrometry Facility for analysis.

#### Tandem mass tag proteomics

Protein lysates were submitted to the UCSD Biomolecular and Proteomics Mass Spectrometry Facility for analysis. Proteins were identified and quantified using Peaks Studio 8.5 (Bioinformatics Solutions Inc.). WebGestalt^65,66^ was used to perform overrepresentation analysis using protein gene symbols with significance ≧13 and abs(log2FC) > 0.3 against a background list of 22,566 protein-coding mouse genes.

### RNA extraction

#### From cells

Frozen cells were processed using a Quick-RNA Miniprep Kit from Zymo Research (R1054) following manufacturers' protocols.

#### From CPMV

In total, 50 μg of CPMV was processed using Quick-RNA Miniprep Kit from Zymo Research (R1054) following manufacturers protocols.

#### From CPMV-infected leaves

CPMV-infected leaves were frozen under liquid nitrogen and then ground with a mortar and pestle until they had a fine texture. Crushed leaves were resuspended in 0.1 M potassium phosphate buffer (pH 7) and filtered through a layer of Mira cloth. Plant sap was treated with 10% SDS and incubated at 50 °C for 10 min. Two volumes of phenol:chloroform:isoamylalcohol (PCI) were added for RNA extractions and vortexed for 1 min, followed by centrifugation at 13,000×*g* for 5 min. The extract was purified using a Quick-RNA Miniprep Kit from Zymo Research (R1054) following manufacturer protocols.

#### RNA transfection

RNA transfection experiments were carried out using Lipofectamine™ MessengerMAX™ Transfection Reagent (Thermo Scientific, LMRNA015) following manufacturers protocols. CleanCap® EGFP mRNA (TriLink Biotechnologies, L-7601-100) was used as a positive control for experiments.

#### RT-PCR

The following primers were designed by Integrated DNA Technologies (IDT). In total, 100 ng of RNA samples were used for RT-PCR experiments. Experiments were performed using Invitrogen SuperScript IV one-step RT-PCR (cat. No. 12594100) following manufacturer protocols. Quantitative RT-PCR was performed using Applied Biosystems TaqMan Fast Virus One Step Master Mix (Thermo Scientific, 4444432) following manufacturer protocols. *Negative strand detection*: Tagged RNA were adapted from primer designs established by others^59,60^. The first strands were synthesized using tagged primers and Invitrogen SuperScript IV First-Strand Synthesis System (Thermo Fisher Scientific, 18-091-050). DNA was then amplified using Invitrogen SuperScript IV one-step RT-PCR (cat. No. 12594100) following manufacturer protocols. Reactions were run on BioRad CFX96 touch real-time PCR detection system.

**Table:** 

**Target**		**Primer Sequence (5**′ **to 3**′**)**
CPMV RNA-1	Forward	AAT CTT CTT GCG GAC GGG AG
	Reverse	CTC TGT GCA TTG TCC TTT TCA CC
CPMV RNA-2 (quantitative)	Forward	GGT ATA GGT TCT AAT CCG GGT ATT G
	Reverse	CAT GGG CTA TAC ACA TCT GAG G
	Probe	56-FAM/TATAGCTCC/ZEN/AAGCAAGCGGGAACC/3IABkFQ
CPMV RNA-2	Forward	GGT TCC CGC TTG CTT GGA GC
	Reverse	GGA GGA TTA TAA ATG TGC G
Negative CPMV RNA-1	Tagged Reverse	**ggc agt atc gtg aat tcg atg c**GG TGT CTC GCT ATC TTG AGT ATG
	Forward	GGC AGT ATC GTG AAT TCG ATG C
	Reverse	CAA CAA GAG CGG GAA CAA ATC
Negative CPMV RNA-2	Tagged Reverse	**tca tgg tgg cga atc c**CG TGC CAA AGA AGG GAA TAA AC
	Forward	TCA TGG TGG CGA ATC C
	Reverse	GTT GAC CAA GCA GTG ACA AAC

#### In vitro transcription of CPMV RNA-2

The plasmid constructs for in vitro transcription of defined CPMV RNA2 sequences were designed as follows. The sequence coding for CPMV RNA2 polyprotein 2 was extracted from NCBI with the accession number MT815985.1. The synthetic construct was retrieved from GenScript and was subcloned in the commercial EGFP vector backbone (BPK1098; Addgene Plasmid #133962) downstream of the T7 promoter, and unique restriction enzymes- BsIWI and *Asc*I were included at the C-terminal. The FLAG sequences (24 bp) were added downstream of the CPMV RNA2 sequence, and polyadenylated tailing (30 bp) was included at the 3′ end. The resulting plasmid cassettes were named pCMV-T7-CPMVRNA2 (Supplementary Data 5c). Linear plasmid DNA template was generated by single digestion using appropriate restriction enzymes, either *BsiW*I or *Asc*I. The in vitro transcripts of CPMV RNA2 were synthesized using HiScribe® T7 ARCA mRNA Kit (NEB) following the manufacturer’s instructions. Purification of the transcribed mRNAs was performed using Lithium chloride solution and was stored at −80 °C. The products of each transcription reaction were analyzed on an Agilent 2100 bioanalyzer and the purity was assessed by UV–Vis Spectroscopy (Supplementary Data 5d).

#### RNA Fluorescent in situ Hybridization multiplexed with Immunofluorescence

Cells were grown on 18 mm glass slides in a 12-well plate. In total, 10^7^ CPMV particles per cell were incubated with cells for 24 h and then washed three times with 1× Phosphate Buffer Solution (Corning, 21-040-CV). Fresh DMEM was added to the cells and maintained until harvest. At each time point, cells were fixed in 7% paraformaldehyde for 2 h at RT, then washed with 1× PBS before permeabilization in 70% ethanol overnight at 4 °C. Cells were blocked in 3% BSA for 1 h at RT, then washed with 1× PBS. For primary antibody incubation, 1 μg antibody/1 mL solution of Rabbit anti-CPMV (Pacific Immunology, 12273/12274) was incubated in 3% BSA in 1× PBS with 0.1% tween for 1 h and rinsed. For secondary incubation, 1:2500 Mouse anti-LAMP-1 Alexa 488 (eBioscience) + 1:2500 Goat anti-Rabbit Alexa 555 (Invitrogen) was incubated in 3% BSA in 1× PBS with 0.1% tween for 1 h and rinsed. Cells were fixed again in 4% paraformaldehyde in 1× PBS for 10 min at RT. Cells were incubated in 10% deionized formamide in 2× SSC buffer for 10 min at RT before RNA hybridization. Custom Stellaris^TM^ probes were designed against CPMV RNAs. These probes were designed using the Stellaris RNA FISH designer (Biosearch Technologies, LGC, Petaluma CA). The probes were labeled with Quasar 647 dye. RNA hybridization was carried out overnight at 37 °C according to manufacturer protocols available at www.biosearchtech.com/stellarisprotocols. Cells were incubated in 10% deionized formamide in 2× SSC for 30 min at 37 °C twice before being mounted on glass slips with Fluoroshield™ with DAPI. Images were taken with a 60× oil objective confocal microscope.

#### Research donor blood

Human blood was obtained from de-identified healthy donor volunteers under the IRB-approved NCI-Frederick Protocol OH99-C-N046. The blood was processed within 2 h after collection. The following vacutainers (Becton Dickinson, Franklin Lakes, NJ, USA) were used: potassium EDTA (complement activation assay), sodium citrate (platelet aggregation assay and plasma coagulation), lithium heparin (hemolysis, leukocyte procoagulant activity, human lymphocyte activation assays). All ethical regulations relevant to human research participants were followed.

#### Selection of concentrations for in vitro hematology and immunology assays

The intended human dose is 10 mg CPMV per tumor. Approximate adult human body weight (70 kg) and blood volume (5.6 L or 8% of body weight) were used to estimate a theoretical human dose (0.14 mg/kg) and theoretical plasma concentration of 2 μg/mL blood. The FDA guidance for the estimation of safe starting human dose was considered^67^. This calculation also assumed that all of the injected dose enters systemic circulation. Thus, the in vitro assays tested the theoretical plasma concentration (2 μg/mL), a 10× concentration (20 μg/mL), and two serial 5-fold dilutions of the theoretical plasma concentration (0.4 μg/mL and 0.08 μg/mL). This estimation is derived from mathematical calculations and assumptions and may differ from the actual CPMV concentrations in the blood under in vivo conditions.

#### Hemolysis

Analysis of CPMV hemolytic properties was performed according to the Nanotechnology Characterization Laboratory (NCL) protocol ITA-1^68^. Briefly, controls and test samples were incubated with human whole blood diluted to the total hemoglobin concentration of 10 mg/mL for 3 h at 37 C, and plasma-free hemoglobin indicative of erythrocyte damage was detected by converting hemoglobin and its metabolites into cyanmethemoglobin (CMH) using Drabkin’s reagent. CMH was then quantified against a hemoglobin standard by measuring the absorbance of the samples at 540 nm using a Spectramax M5 plate reader and SoftmaxPro software (Molecular Devices, San Jose, CA, USA).

#### Platelet aggregation

The analysis of CPMV ability to induce platelet aggregation or affect collagen-induced platelet aggregation was performed according to the NCL protocol ITA-2.1 (https://www.cancer.gov/nano/research/ncl/protocols-capabilities/ncl-method-ita-2.1.pdf)^69^. In brief, platelet-rich plasma (PRP) and platelet-poor plasma (PPP) were prepared from freshly drawn human blood. Plasma from three donors was pooled. PPP was used as the background control. PRP was incubated with test samples, and a number of single platelets was counted using a Z2 analyzer (Beckman Coulter, Brea, CA, USA). Platelet-poor plasma combined with nanoparticles was used to monitor potential particle aggregation or agglomeration in order to rule out false-negative results. The percent platelet aggregation was calculated by comparing the number of single (unaggregated) platelets in the negative control group with that in the test sample.

#### Plasma coagulation

CPMV effects on human plasma coagulation were studied following NCL protocol ITA-12, (https://www.cancer.gov/nano/research/ncl/protocols-capabilities/ncl-method-ita-12.pdf)^69^. Three plasma coagulation tests were performed: prothrombin time (PT), activated partial thromboplastin time (APTT), and thrombin time (TT), corresponding to the extrinsic, intrinsic, and common pathways, respectively. Briefly, freshly drawn human blood from three donors was used to prepare pooled plasma. The pooled plasma was then incubated with test samples for 30 min at 37 °C. Following incubation, plasma coagulation initiation reagents (neoplastin, CaCl_2_, or thrombin, respectively) were added to the mixture, and the coagulation times were measured using the STArt4 coagulometer (Stago-US, Parsippany, NJ, USA).

#### Complement activation

To analyze complement activation, NCL protocol ITA-5.2 (https://www.cancer.gov/nano/research/ncl/protocols-capabilities/ncl-method-ita-5.2.pdf) was followed^70^. Briefly, plasma was prepared from freshly drawn human blood. Plasma from three donors was pooled and incubated with test samples and veronal buffer for 30 min at 37 °C. Following incubation, the samples were analyzed for the presence of the iC3b component of complement using a commercial enzyme immunoassay kit (Quidel, San Diego, CA, USA). Cremophor was included as an additional control and was tested at the concentration that mimics that in the commercial formulation Taxol, which is known to cause complement-mediated toxicity in patients^71^.

#### Chemotaxis

Experiments were performed according to the NCL protocol ITA-8.1 (https://www.cancer.gov/nano/research/ncl/protocols-capabilities/ncl-method-ita-8.1.pdf)^72^. Briefly, test samples were deposited into wells of the bottom compartment of a MultiScreen filter plate MAMCS9610 (Millipore, Burlington, MA, USA). Model phagocytes (the promyelocytic leukemia cell line, HL-60), starved overnight before the experiment, were then placed into the wells of the top compartment of these plates. A 3 µm filter at the bottom of the top compartment plate separates the cells from the controls and test samples in the bottom compartment. The cells from the top plate migrate through the filter if the sample in the corresponding well of the bottom plate has chemoattractive properties. After 4 h of incubation, the migrated cells were detected using fluorescent dye Calcein AM using Spectramax M5 plate reader and SoftmaxPro software (Molecular Devices, San Jose, CA, USA).

#### Phagocytosis

Experiments were performed according to the NCL protocol ITA-9.2 (https://www.cancer.gov/nano/research/ncl/protocols-capabilities/ncl-method-ita-9.2.pdf)^73^. Briefly, model phagocytes (promyelocytic leukemia cell line HL-60) were treated overnight with control or CPMV particles. The cell viability was then estimated with a Cellometer using an acridine orange and propidium iodide (AO/PI) dye (Nexcelome Bioscience LLC., Lawrence, MA, USA). Next, the cells were incubated for 2 h with opsonized zymosan A in the presence of luminol. The intensity of the luminescent signal proportional to the number of zymosan particles taken up by the cells was recorded every 2.5 min over a 2-h time frame using Spectramax M5 plate reader and SoftmaxPro software (Molecular Devices, San Jose, CA, USA). The phagocytic activity of cells in each test sample was then estimated by calculating the area under the curve (AUC).

#### Human lymphocyte activation (HuLa)

The study was performed according to the NCL protocol ITA-18^74^. Briefly, peripheral blood mononuclear cells (PBMC) were collected from three healthy donor volunteers vaccinated with flu vaccine. PBMC were incubated with test samples and controls for 1 h before stimulation with flu haemagglutinin from three viral strains present in the seasonal vaccine mix (A/California/07/2009 X-179A (H1N1), A/Switzerland/9715293/2013 NIB-88 (H3N2), and B/Phuket/3073/2013 (B. Yamagata lineage)). Following stimulation, the incubation was continued for an additional 72 h. At the end of the incubation period, BrdU was added to the cells, and incubated for an additional 24 h. The BrdU incorporated into the DNA of proliferating cells was detected using a commercial ELISA kit according to the manufacturer’s instructions (Sigma-Aldrich, St.Louis, MO, USA).

#### Leukocyte procoagulant activity (PCA)

The experiment was conducted according to the NCL protocol ITA-17^69^. Briefly, PBMC was isolated from healthy human donors and incubated with controls and test samples for 24 h at 37 °C. Duplicate samples were prepared for each treatment. The treated cells were washed twice with PBS to remove CPMV and resuspended in calcium chloride buffer. The resuspended samples were then added to autologous plasma to test for induction of plasma coagulation. Coagulation was measured using the Start4 coagulometer (Stago-US, Parsippany, NJ, USA). The formulations were compared to the positive control consisting of K12 *E.Coli* lipopolysaccharide (LPS) and calcium-ionophore (Ca-Iono).

#### Statistics and reproducibility

Generated data was analyzed using GraphPad Prism with a sample size or replication of at least 2. The mean and standard deviation was plotted on the graphs. The replicates and sample size for individual experiments are included in respective figure legend.

### Core facilities

#### Electron microscopy core

The authors thank the University of California, San Diego—Cellular and Molecular Medicine Electron Microscopy Core (UCSD-CMM-EM Core, RRID: SCR_022039) for equipment access and technical assistance. The UCSD-CMM-EM Core is partly supported by the National Institutes of Health Award number S10OD023527.

#### Biomolecular and proteomics mass spectrometry facility

The authors acknowledge Majid Ghassemian of the Biomolecular and Proteomics Mass Spectrometry Facility (BPMSF) at the University of California, San Diego, for his assistance and use of facilities. The BPMSF is funded by the NIH under Grants S10 OD016234 (Synapt-HDX-MS) and S10 OD021724 (LUMOS Orbi-Trap).

#### MCC confocal microscopy

The authors thank Kersi Pestonjamasp and acknowledge the UCSD Cancer Center Support Grant 2P30CA023100 by NCI.

#### Center for computational biology and bioinformatics (CCBB)

Altman clinical and Translational Institute (ACTR) grant # UL1TR001442.

#### IGM genomics center

This publication includes data generated at the UC San Diego IGM Genomics Center utilizing an Illumina NovaSeq 6000 that was purchased with funding from a National Institutes of Health SIG grant (#S10 OD026929).

#### UCSF Chimera software

Molecular graphics and analyses were performed with UCSF Chimera, developed by the Resource for Biocomputing, Visualization, and Informatics at the University of California, San Francisco, with support from NIH P41-GM103311.

### Reporting summary

Further information on research design is available in the Nature Portfolio Reporting Summary linked to this article.

## Supplementary information

Supplementary information

Description of Additional Supplementary File

Supplementary table 1

Supplementary Movie 1

Reporting summary

## Data Availability

Data points for the volcano and gene ontology figures are attached as supporting files. All other datasets that support this study are publicly available at the National Cancer Institute’s Center for Biomedical Informatics and Information Technology public repository (https://cananolab.cancer.gov). RNA sequencing data is available at NCBI under accession ID: PRJNA1150650.
