# Supplementary material for: Cellular fate of a plant virus immunotherapy candidate
Source: Commun Biol. 2024 Oct 24;7:1382. doi: 10.1038/s42003-024-06982-0 (PMC11499861; doi:10.1038/s42003-024-06982-0)
Supplement: Supplementary file 1 — Supplementary information [file 42003_2024_6982_MOESM1_ESM.pdf]

SUPPORTING INFORMATION

Cellular fate of a plant virus immunotherapy candidate

Anthony O. Omole[1-4], Jessica Fernanda Affonso de Oliveira[1-4], Lucas Sutorus[1-4], Sweta Karan[1-4], Zhongchao Zhao[1-4], Barry W. Neun[9], Edward Cedrone[9], Jeffrey D. Clogston[9], Jie Xu[9], Michael Sierk[10], Qingrong Chen[10], Daoud Meerzaman[10], Marina A. Dobrovolskaia[9], Nicole F. Steinmetz*[1-8]

[1]Aiiso Yufeng Li Family Department of Chemical and Nano Engineering, University of California, San Diego, La Jolla, CA, United States
[2]Shu and K.C. Chien and Peter Farrell Collaboratory, University of California, San Diego, La Jolla, CA, USA
[3]Center for Nano-ImmunoEngineering, University of California, San Diego, La Jolla, CA, United States
[4] Moores Cancer Center, University of California, San Diego, La Jolla, CA, United States
[5]Department of Bioengineering, University of California, San Diego, La Jolla, CA, United States
[6]Department of Radiology, University of California, San Diego, La Jolla, CA, United States
[7]Institute for Materials Discovery and Design, University of California, San Diego, La Jolla, CA, United States
[8]Center for Engineering in Cancer, Institute of Engineering Medicine, University of California, San Diego, La Jolla, CA, United States
[9]Nanotechnology Characterization Lab, Cancer Research Technology Program, Frederick National Laboratory for Cancer Research sponsored by the National Cancer Institute, Frederick, MD, United States
[10]Center for Biomedical Informatics and Information Technology, National Cancer Institute, National Institutes of Health, Bethesda, MD, United States

* Corresponding author: nsteinmetz@ucsd.edu

ORCID: https://orcid.org/0000-0002-0130-0481

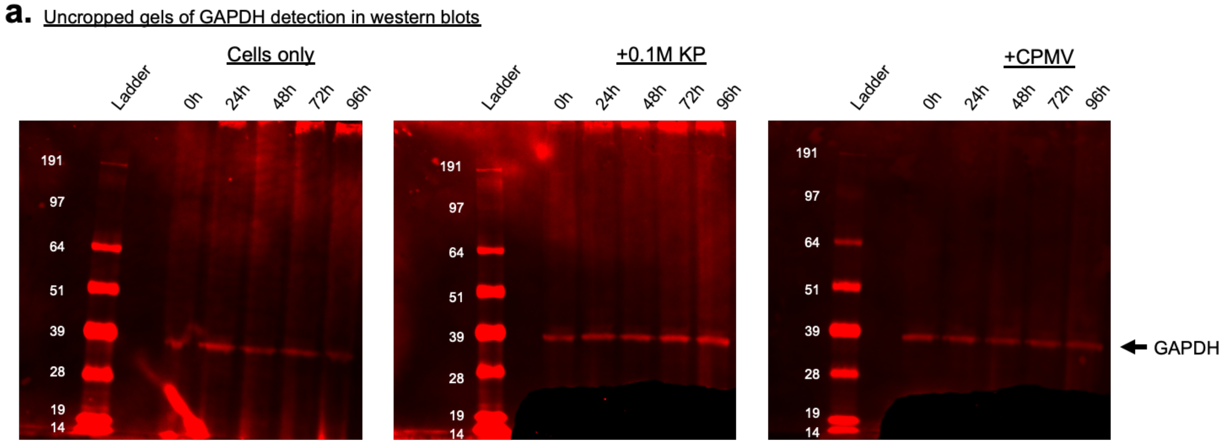

Supplementary Data 1 *a) uncropped gels of GAPDH detection*

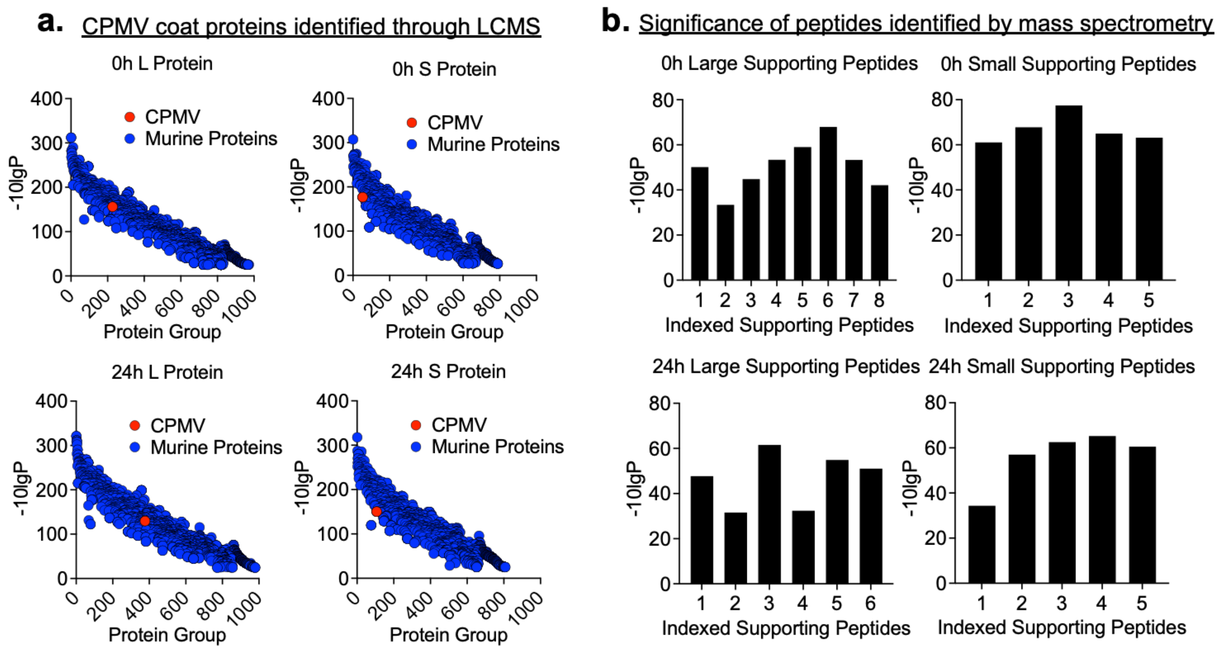

Supplementary Data 2 *(a) Panel shows CPMV coat proteins identified among RAW 264.7 macrophage proteins. (b) Significance of CPMV coat protein peptides detected that support CPMV detection.*

Peptide location	Indexed supporting peptide sequence
Small CP 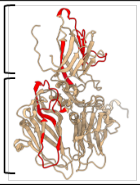 Large CP	MEQNLFALSL DDTSSVRGSL LDTKFAQTRV LLSKAMAGGD VLLDEYLYDV VNGQDFRATV AFLRTHVITG KIKVTATTNI SDNSGCCLML AINSGVRGKY STDVYTICSQ DSMTWNPGCK KNFSFTFNPN PCGDSWSAEM ISRSRVRMTV ICVSGWTLSP TTDVIAKLDW SIVNEKCEPT IYHLADCQNW LPLNRWMGK[1]**L TFPQGVTSEV R**RM[2]**PLSIGGG AG**ATQAFLAN MPNSWISMWR YFRGELHFEV TKMSSPYIKA TVTFLIAFGN LSDAFGFYES FPHR[3]**IVQFAE VEEK**CTLVFS QQEFVTAWST QVNPRTTLEA DGCPYLYAII HDSTTGTISG DFNLGVK[4]**LVG IKDFCGIGSN PGIDGSR**LLG AIAQGPVCAE ASDVYSPCMI ASTPPAPFSD VTAVTFDLIN GKITPVGDDN WNTHIYNPPI MNVLRTAAWK [5]**SGTIHVQLNV R**GAGVK[6]**RADW DGQVFVYLR**[7]**Q SMNPESYDAR** TFVISQPGSA MLNFSFDIIG PNSGFEFAES PWANQTTW[8]**YL ECVATNPR**QI QQFEVNMRFD PNFRVAGNIL MPPFPLSTET PPLLKFRFRDI ERSKRSVMVG HTATAA
Small CP 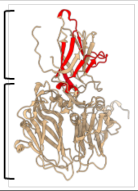 Large CP	MEQNLFALSL DDTSSVRGSL LDTKFAQTRV LLSKAMAGGD VLLDEYLYDV VNGQDFRATV AFLRTHVITG KIKVTATTNI SDNSGCCLML AINSGVRGKY STDVYTICSQ DSMTWNPGCK KNFSFTFNPN PCGDSWSAEM ISRSRVRMTV ICVSGWTLSP TTDVIAKLDW SIVNEKCEPT IYHLADCQNW LPLNRWMGKL TFPQGVTSEV RRMPLSIGGG AGATQAFLAN MPNSWISMWR YFRGELHFEV TKMSSPYIKA TVTFLIAFGN LSDAFGFYES FPHRIVQFAE VEEKCTLVFS QQEFVTAWST QVNPRTTLEA DGCPYLYAII HDSTTGTISG DFNLGVKLVG IKDFCGIGSN PGIDGSRLLG AIAQGPVCAE ASDVYSPCMI ASTPPAPFSD VTAVTFDLIN GK[1]**ITPVGDDN WNTHIYNPPI MNVLR**TAAWK [2]**SGTIHVQLNV R**GAGVK[3]**RADW DGQVFVYLR**[4]**Q SMNPESYDAR** TFVISQPGSA MLNFSFDIIG PNSGFEFAES PWANQTTWYL ECVATNPR[5]**QI QQFEVNMR**FD PNFRVAGNIL MPPFPLSTET PPLLKFRFRDI ERSKRSVMVG HTATAA
Small CP 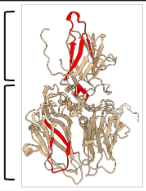 Large CP	MEQNLFALSL DDTSSVRGSL LDTKFAQTRV LLSKAMAGGD VLLDEYLYDV VNGQDFRATV AFLRTHVITG KIKVTATTNI SDNSGCCLML AINSGVRGKY STDVYTICSQ DSMTWNPGCK KNFSFTFNPN PCGDSWSAEM ISRSRVRMTV ICVSGWTLSP TTDVIAKLDW SIVNEKCEPT IYHLADCQNW LPLNRWMGK[1]**L TFPQGVTSEV R**RMPLSIGGG AGATQAFLAN MPNSWISMWR YFR[2]**GELHFEV TK**MSSPYIKA TVTFLIAFGN LSDAFGFYES FPHRIVQFAE VEEKCTLVFS QQEFVTAWST QVNPRTTLEA DGCPYLYAII HDSTTGTISG DFNLGVKLVG IKDFCGIGSN PGIDGSRLLG AIAQGPVCAE ASDVYSPCMI ASTPPAPFSD VTAVTFDLIN GKITPVGDDN WNTHIYNPPI [3]**MNVLRTAAWK S**GTIHV[4]**QLNV RGAGVKRAD**[5]**W DGQVFVYLRQ** SMNPESYDAR TFVISQPGSA MLNFSFDIIG PNSGFEFAES PWANQTTWYL ECVATNPR[6]**QI QQFEVNMR**FD PNFRVAGNIL MPPFPLSTET PPLLKFRFRDI ERSKRSVMVG HTATAA
Small CP 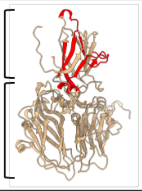 Large CP	MEQNLFALSL DDTSSVRGSL LDTKFAQTRV LLSKAMAGGD VLLDEYLYDV VNGQDFRATV AFLRTHVITG KIKVTATTNI SDNSGCCLML AINSGVRGKY STDVYTICSQ DSMTWNPGCK KNFSFTFNPN PCGDSWSAEM ISRSRVRMTV ICVSGWTLSP TTDVIAKLDW SIVNEKCEPT IYHLADCQNW LPLNRWMGKL TFPQGVTSEV RRMPLSIGGG AGATQAFLAN MPNSWISMWR YFRGELHFEV TKMSSPYIKA TVTFLIAFGN LSDAFGFYES FPHRIVQFAE VEEKCTLVFS QQEFVTAWST QVNPRTTLEA DGCPYLYAII HDSTTGTISG DFNLGVKLVG IKDFCGIGSN PGIDGSRLLG AIAQGPVCAE ASDVYSPCMI ASTPPAPFSD VTAVTFDLIN GKI[1]**TPVGDDN WNTHIYNPPI MNVLR**TAAWK [2]**SGTIHVQLNV R**GAGVK[3]**RADW DGQVFVYLR**[4]**Q SMNPESYDAR** TFVISQPGSA MLNFSFDIIG PNSGFEFAES PWANQTTWYL ECVATNPR[5]**QI QQFEVNMR**FD PNFRVAGNIL MPPFPLSTET PPLLKFRFRDI ERSKRSVMVG HTATAA

Supplementary Data 3 *Supporting peptide location and sequence on CPMV coat protein. Highlighted Glycine shows start of small coat protein.*

a. Gating strategy for flow cytometry detection of CPMV-Cy5 in cells

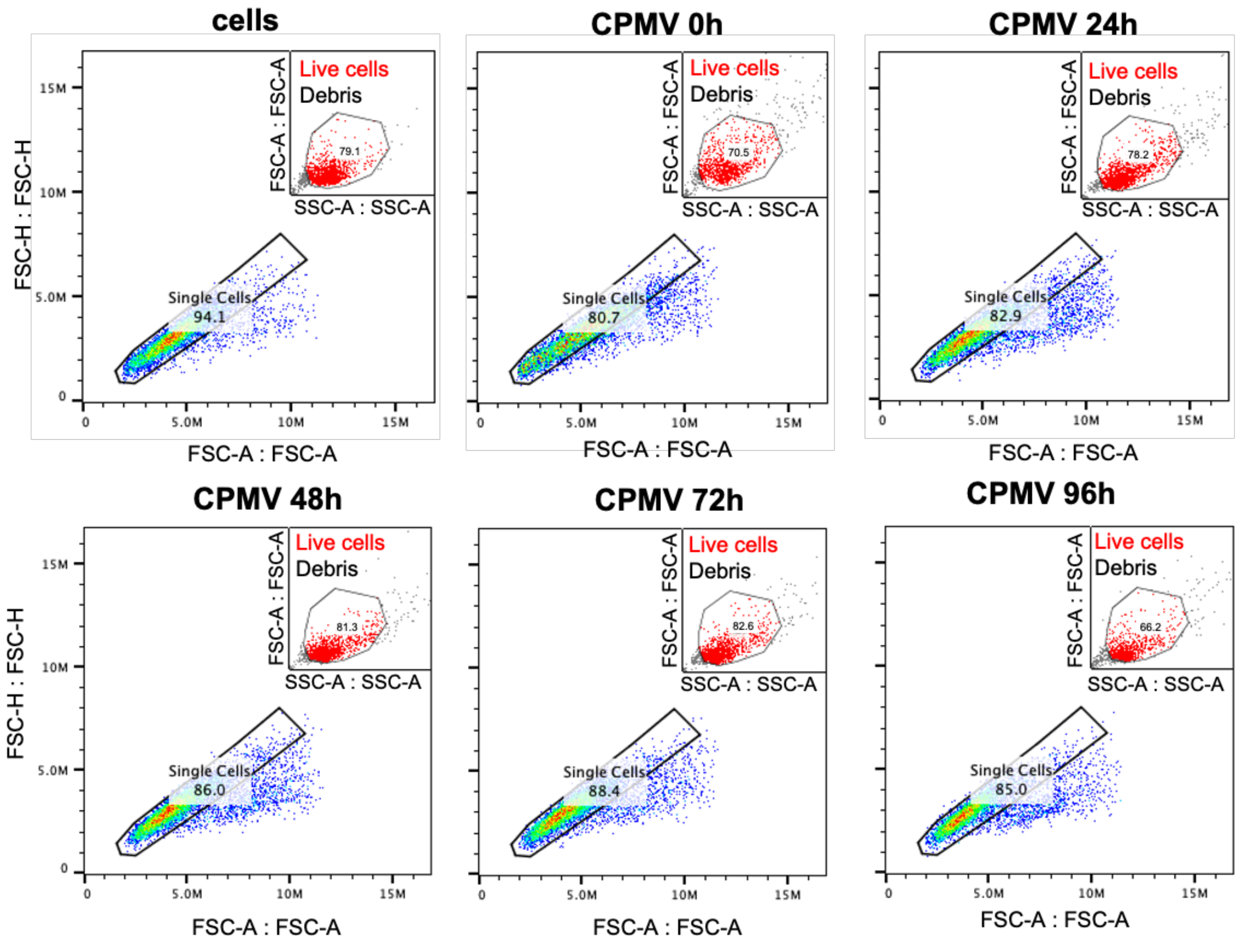

Supplementary Data 4 *Gating strategy for CPMV-Cy5 detection in cells.*

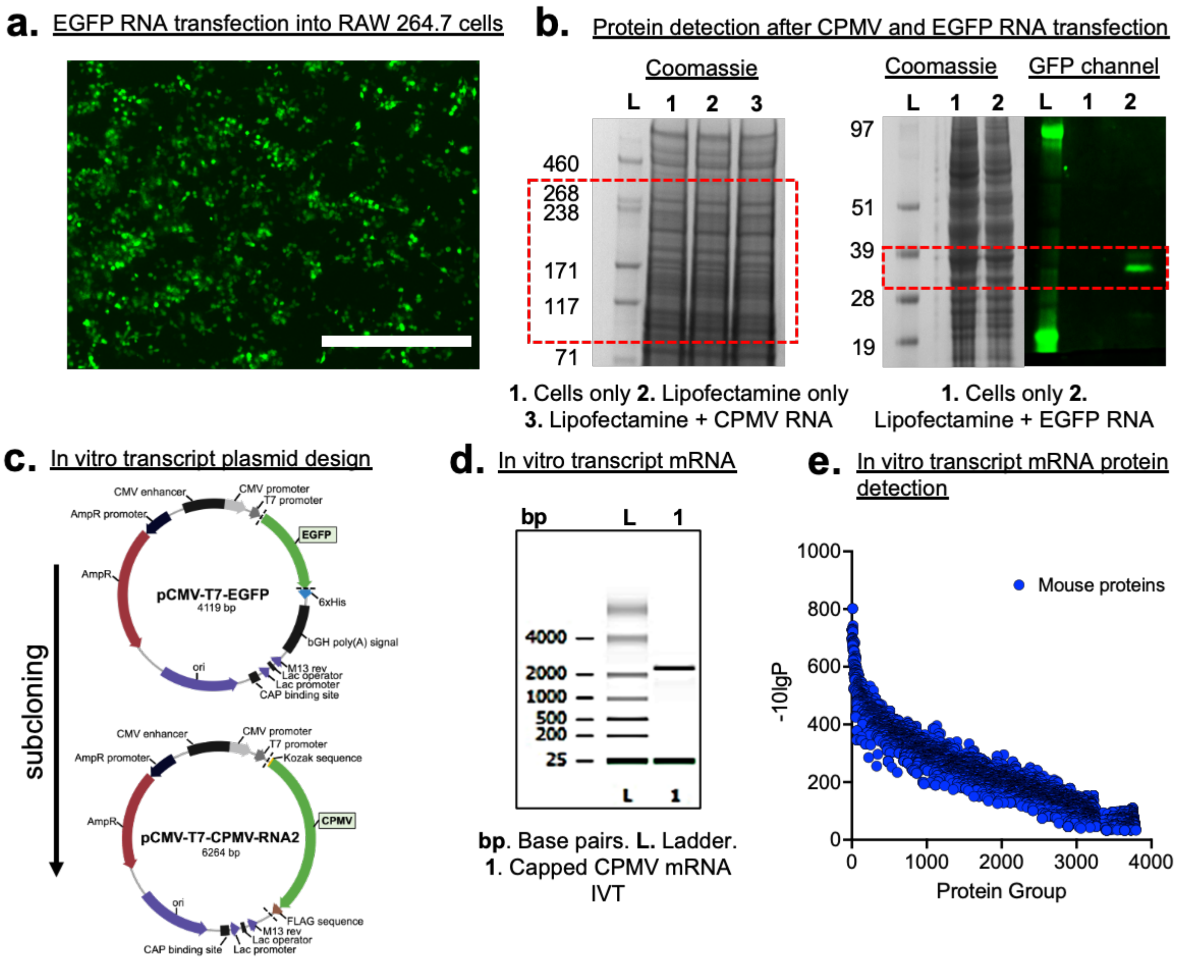

Supplementary Data 5 *(a) GFP expression is evident after transfection of GFP RNA. Scale bar to 400 □M. (b) CPMV related protein not evident on SDS gel while GFP expression can be detected by on SDS gel. (c) Subcloning of CPMV RNA plasmid from pCMV-T7-EGFP. (d) Size detection of IVT CPMV RNA-2 is ~2865 bp. (e) No MudPIT detection of CPMV RNA-2 polyprotein after lipofectamine-assisted transfection.*

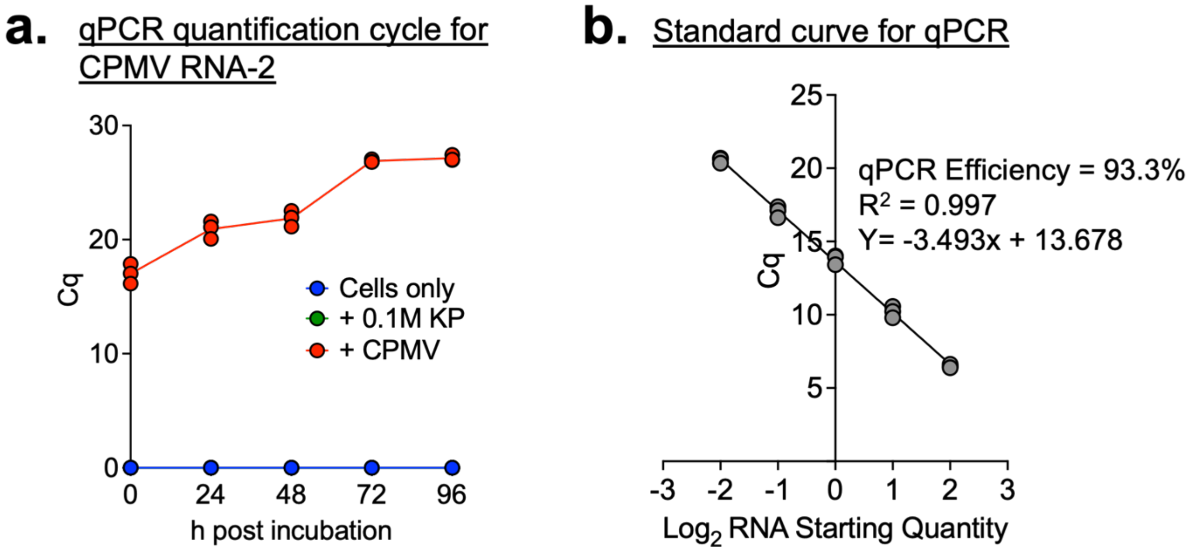

Supplementary Data 6 *(a) Quantification cycle for CPMV RNA-2 detection in cells. N=3 (b) Standard curve for CPMV RNA-2 detection;* R^2 *= 0.997 and qPCR efficiency is 93.3%. N=3.*

a. Uncropped gels of standard RT-PCR detection of negative sense CPMV RNA-1 and CPMV RNA-2

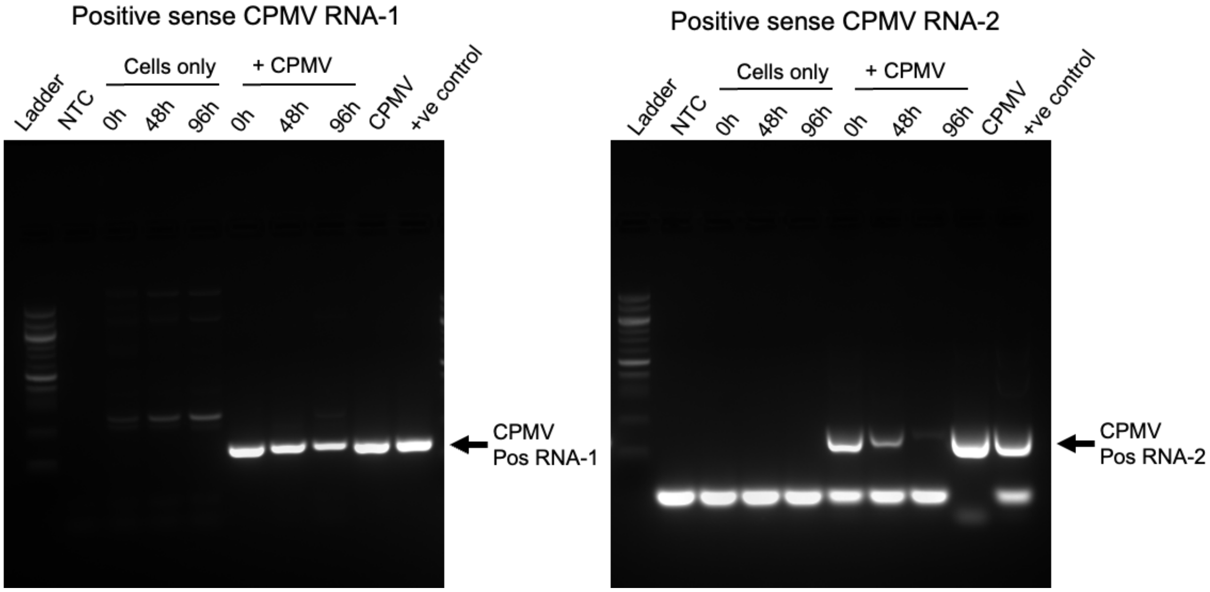

***Supplementary Data 7**: a) Uncropped gels for CPMV RNA-1 and RNA-2 negative sense detection.*

Supplementary Movie 1: See video attached. Multiplexed confocal imaging with RNA fluorescent *in situ* hybridization after CPMV RNA transfection. Red: cell membrane, Blue: DAPI (Nucleus), Yellow: LAMP-1, Green: CPMV RNAs

Supplementary Table 1: See table attached. Full list of upregulated and downregulated genes and gene ontological processes.
