# Supplementary material for: Cellular fate of a plant virus immunotherapy candidate
Source: Commun Biol. 2024 Oct 24;7:1382. doi: 10.1038/s42003-024-06982-0 (PMC11499861; doi:10.1038/s42003-024-06982-0)
Supplement: Supplementary file 2 — Description of Additional Supplementary File [file 42003_2024_6982_MOESM2_ESM.pdf]

Description Of Additional Supplementary File

File name: Supplementary Data 1
Description: Full list of genes upregulated and downregulated + Full list of gene ontological process

File name: Supplementary Movie 1
Description: A video showing the transfection of viral RNA
